# Antibacterial, Antifungal, Antimycotoxigenic, and Antioxidant Activities of Essential Oils: An Updated Review

**DOI:** 10.3390/molecules25204711

**Published:** 2020-10-14

**Authors:** Aysegul Mutlu-Ingok, Dilara Devecioglu, Dilara Nur Dikmetas, Funda Karbancioglu-Guler, Esra Capanoglu

**Affiliations:** 1 Department of Food Processing, Akcakoca Vocational School, Duzce University, 81650 Akcakoca, Duzce, Turkey; aysegulmutlu@duzce.edu.tr; 2 Department of Food Engineering, Faculty of Chemical and Metallurgical Engineering, Istanbul Technical University, 34469 Maslak, Istanbul, Turkey; devecioglud@itu.edu.tr (D.D.); dikmetas@itu.edu.tr (D.N.D.)

**Keywords:** essential oils, antibacterial, antifungal, antimycotoxigenic, antioxidant

## Abstract

The interest in using natural antimicrobials instead of chemical preservatives in food products has been increasing in recent years. In regard to this, essential oils—natural and liquid secondary plant metabolites—are gaining importance for their use in the protection of foods, since they are accepted as safe and healthy. Although research studies indicate that the antibacterial and antioxidant activities of essential oils (EOs) are more common compared to other biological activities, specific concerns have led scientists to investigate the areas that are still in need of research. To the best of our knowledge, there is no review paper in which antifungal and especially antimycotoxigenic effects are compiled. Further, the low stability of essential oils under environmental conditions such as temperature and light has forced scientists to develop and use recent approaches such as encapsulation, coating, use in edible films, etc. This review provides an overview of the current literature on essential oils mainly on antifungal and antimycotoxigenic but also their antibacterial and antioxidant activities. Additionally, the recent applications of EOs including encapsulation, edible coatings, and active packaging are outlined.

## 1. Introduction

The consumer’s tendency to buy more natural and less processed food products encouraged the food industry to use natural alternatives [1]. This situation has powered scientists to investigate the novel natural substances of medicinal and aromatic plants [2]. Essential oils (EOs) are secondary metabolites of plants that have volatile, natural, and complex characteristics [3]. The positive health effects of EOs extracted from aromatic and medicinal plants have been known since ancient times. Several EOs and metabolites in plant extracts are accepted as “Generally Recognised as Safe” (GRAS) [4]. Antimicrobial and antioxidant activities of EOs have been investigated in several studies, and these studies have shown antimicrobial and antioxidant activities as the most common biological activities in EOs, but some other activities of EOs, including antiviral, insecticidal, angiotensin-converting enzyme, amylase and glucosidase enzyme, and xanthine oxidase inhibitory activities, etc., still need more investigation. In addition to their individual use, EO combinations can be used in binary or ternary mixtures to improve the mixtures’ biological activities [5].

Food products are often contaminated not only with pathogen microorganisms but also with molds and the toxins produced by these molds. This contamination can be encountered at different stages of the food chain such as post-harvest processing, transportation, and storage stages. Similar to bacterial contamination, fungal growth and mycotoxins lead to quality and quantity defects and loss of market value in addition to their health risks. Additionally, mold invasion leads to noticeable quality and organoleptic changes (e.g., off-flavors, defects in texture and color) [6]. In addition to their health risks, food losses due to microbiological contamination should also be considered, especially when the increasing world population and food requirements are taken into account. According to the Food and Agricultural Organization of the United Nations (FAO), foodborne molds and their toxic metabolites cause about 25% loss in agricultural foodstuffs worldwide [7]. On the other hand, some fungal genera (e.g., *Fusarium*, *Penicillium*, *Aspergillus*, and *Alternaria*) may produce secondary metabolites, called mycotoxins. Some of these mycotoxins may be lethal and they may have carcinogenic, mutagenic, teratogenic, and immunosuppressive effects on humans and animals. Nonetheless, inhibition of the growth of mycotoxigenic fungi is not sufficient to obtain the safety of food, but toxin reduction should also be considered and investigated [8]. As a result, researchers have focused on how to create cost-effective and economic natural preservatives to control both microbial contamination and mycotoxin production [9].

In vitro studies have been followed by in vivo antimicrobial tests to measure the efficacy of EOs in the food systems. There are studies focusing on the application of EOs especially using recent technologies such as edible coating on beef [10] and on cheese [11] and nanoencapsulation for bread [12]. In addition to their usage as antimicrobial agents, their antioxidant activities have also received great interest. Since EOs are recognized as natural antioxidants, and they mostly have a non-toxic nature, studies on EOs have attracted more attention for their potential use in place of synthetic antioxidants. Recently, many research studies have been carried out on the antioxidant activity of different EOs, and most of these studies put antioxidant activity of EOs at the forefront [13].

The function of secondary metabolites from medicinal and aromatic plants, which can be in the structure of terpenes, phenols, aldehydes, esters, alcohols, and ketones, are strongly correlated with their biochemical and physiological features [3]. The above-mentioned biological activities of EOs are generally associated not only with major components but also minor components of EOs [14]. The chemical compositions of plant EOs vary depending on the type of the plant species and their geographical location, environment, and maturation stage, as well as the obtaining method of EOs [15]. Additionally, the part of the plant where the EO is obtained from including flowers, stems, leaves, or buds is another critical factor [16]. To the best of our knowledge, while their antibacterial and antioxidant activities are well known, antifungal and antimycotoxigenic activities of EOs have yet to be investigated deeply. In addition to all these facts, some strategies are required to protect the biological activities of EOs during food processing. EOs are volatile at room temperature [17] and can be easily degraded by oxygen and temperature fluctuations [18]. Therefore, methods to increase the stability and activity of EOs are required, and novel techniques such as encapsulation, the use of edible coatings, and active packaging may provide the opportunity to solve these problems [19].

This review provides a comprehensive literature review about EOs that are significantly active against fungi and mycotoxins as well as providing a perspective on the antibacterial and antioxidant activities of several EOs. Recent applications of EOs including the encapsulation or edible coatings are also explained in detail. Finally, the future possibilities in the use of essential oils derived from aromatic and medicinal plants are discussed.

## 2. Antimicrobial Activities of Essential Oils

Essential oils have antimicrobial and other biological activities due to their bioactive volatile components [20]. Within the compounds present in EOs—such as terpenes, alcohols, acids, esters, epoxides, aldehydes, ketones, amines, and sulfides—terpineol, thujanol, myrcenol, neral, thujone, camphor, and carvone are the most critical ones for their activities [14,21]. Although major components are generally responsible for their biological activities, the contribution of minor components to these activities should not be omitted. The use of different microbial cultures, different EOs, different concentration ranges, and the use of different antimicrobial test methods on different media have led to the formation of an extensive database. Although the antibacterial effect of EOs has been the subject of several studies for many years, the number of studies concerning the antifungal and antimycotoxigenic effects has not yet reached sufficient levels. Increased cases of fungal infections in recent years and the contamination of food and animal feed products with mycotoxins are considerable issues for both consumers and producers. Nowadays, there has been a significant increase in the number of studies performed on other biological activities of EOs because of the increasing data on the cidal or static effects of EOs on microorganisms.

Regarding the mechanism of action, it has been suggested that EOs can affect the cell membrane of bacterial and fungal cultures. The antimicrobial activity of EOs occurs in the way that they can easily disrupt the cell membrane and make it more permeable [14,20]. Moreover, they interrupt ion transport processes and they have interaction with membrane proteins and other compounds within the cell [22,23,24]. EOs also have adverse effects on enzymes by acting on their active sites [25]. A loss of electrolytes was detected after EO treatment as measured by the concentrations of K^+^, Ca^2+^, and Na^+^ ions [26]. In short, antimicrobial effects were found to be associated with the interaction of EO and the cell system, especially against the plasma membrane and the disruption of the functions of mitochondria [27]. An imbalance between intracellular and extracellular ATP concentrations eventually leads to cell death [28]. It was also claimed that the antimicrobial effects of EOs may be related to the diffusion ability of EOs (diffusion coefficient, zeta potential, and droplet size of EOs) through the cell membrane of microorganisms [29]. Although the antimicrobial action mechanisms of EOs were well established in the literature, there is not sufficient information for the action of the antimycotoxigenic mechanism. However, several approaches have been reported in recent works. In one of these studies, the antimycotoxigenic action mechanism of EOs was directly correlated with their influence on the aflatoxin biosynthesis pathway [30].

The use of essential oils at high concentrations in foods may lead to organoleptic problems because of their odorous characteristics. From this point of view, using essential oils in combination is considered as an important approach in terms of reducing the required concentrations to contribute to food safety. The increase in the antibacterial activity of EOs, when used in combination, has been proposed by several researchers. Not only synergistic but also additive, non-interactive, and antagonistic interactions have been reported [5]. Within these interactions, EO combinations that have synergistic and additive effects have been suggested for food applications [31]. The synergism was reported when the EO combination had higher inhibitory effect than the inhibitory activities of individual EOs [32]. In a research investigating the antibacterial activity of thyme and oregano EOs and their major compounds (thymol and carvacrol), the combination of thymol and carvacrol, aromatic oxygenated monoterpenes, were found to have additive antibacterial effects against *Staphylococcus aureus* and *Bacillus cereus*, *Salmonella infantis*, and *Escherichia coli*. Moreover, additive antibacterial action was observed with the combination of thyme and oregano EOs [33]. While no antagonism was reported for the combinations of cardamom, cumin, and dill weed, the highest antimicrobial activity was observed in a cardamom and dill weed EO combination. This high antimicrobial activity was attributed to the synergistic effect of 1,8-cineole content (29.2%) of cardamom EO and limonene content (27.4%) of dill weed EO [5]. A synergistic effect was also reported for the combination of cinnamon (cinnamaldehyde as the main constituent) and clove (eugenol as the main constituent) against *Staphylococcus aureus*, *Listeria monocytogenes*, *Salmonella typhimurium*, and *Pseudomonas aeruginosa* and synergistic antifungal activity against *Aspergillus niger* [34]. As mentioned previously, the efficacy of EOs used in combination was not only tested against bacteria but also against fungi. In this respect, within carvacrol, thymol, *p*-cymene, and 1,8-cineole interactions, the most synergetic combinations were reported to be thymol/1,8-cineole and thymol/*p*-cymene against *Candida* spp. [35]. In another research, antimicrobial activities of oregano, clove, and cinnamon EOs that have aromatic compounds (eugenol in clove and cinnamon EOs and carvacrol in oregano EO) were attributed to the presence of an aromatic nucleus and a phenolic OH group [36].

In this part of the review, we mainly focus on the studies related with the antimicrobial activities of the most common plant EOs. While the antibacterial activities of EOs have been reviewed, especially using recent researches, antifungal and antimycotoxigenic activities have been emphasized more.

### 2.1. Antibacterial Activities of Essential Oils

The antibacterial effects of essential oils occur in two ways: either by the restriction of the bacterial growth (bacteriostatic) or by killing the bacterial cells (bactericidal) [37]. These antibacterial activities can be determined by using agar/disc diffusion, broth micro/macro dilution, and agar dilution methods. The antimicrobial activity of EOs mainly depends on the chemical composition as well as the parts of the plants. On the other hand, Gram-positive and Gram-negative bacteria differ in terms of their sensitivity against EOs [5,37]. These differences have been explained by several mechanisms including the more resistant nature of Gram-negative bacteria due to their double layer of phospholipids [21]. In the meantime, the antibacterial activity of different plant EOs is well-documented against both Gram-positive (*Bacillus subtilis*, *Staphylococcus aureus*, *Listeria monocytogenes*) and Gram-negative (*Escherichia coli*, *Salmonella typhimurium*, *Pseudomonas aeruginosa*, *Camplyobacter* spp.) bacteria [11,38,39,40,41,42]. In addition to these, clinical and standard strains can also differ in terms of their sensitivities against EOs [42]. The most common EOs are listed for their antibacterial activities on a wide range of bacteria, and the studies carried out on this subject in the last five years are summarized in Table 1.

Bouyahya et al. [49] examined the antibacterial activity of steam-distilled *Mentha pulegium* and *Rosmarinus officinalis* EOs that were predominated by oxygenated monoterpenes as 63.7% and 83.9%, respectively, against *Staphylococcus aureus*, *Pseudomanas aeruginosa*, *Listeria monocytogenes*, *Bacillus subtilis*, *Escherichia coli*, and *Proteus mirabilis*. The authors indicated that *M. pulegium* EO was more effective than the EO from *Rosmarinus officinalis* on the tested bacteria. Additionally, when compared with commercial antibiotics, *Mentha plugieum* EO was found to have significant antibacterial activity. In addition to the above-mentioned microorganisms, *Rosmarinus officinalis* was also effective against *Enterococcus faecalis*, *Klebsiella pneumoniae* [48], and *Pseudomanas aeruginosa* [43]. The antibacterial activity of the most widely used EOs from *Thymus* species, which belong to the Lamiaceae family, was also demonstrated against several microorganisms such as *Salmonella typhi*, *Salmonella typhimurium*, *Escherichia coli*, *Staphylococcus aureus*, *Streptococcus pneumonia*, *Bacillus cereus*, *Pseudomanas aeruginosa*, *Proteus mirabilis*, *Klebsiella pneumoniae*, and *Listeria monocytogenes* [16,52,53,54]. On the other hand, the EO from *Origanum vulgare* (oregano) was one of the most effective EOs against microorganisms. Its antibacterial activity against *Salmonella typhimurium*, *Escherichia coli*, *Bacillus cereus*, *Pseudomanas aeruginosa*, and *Enterococcus faecalis* has been reported in several studies [50,55]. There are also studies comparing the antibacterial effect of different EOs. In a study performed by Pesavento et al. [46], the antibacterial activities of EOs from *Rosmarinus officinalis*, *Cinnamomum zeylanicum*, *Thymus vulgaris*, *Origanum vulgare*, and *Salvia officinalis* were compared, and *Thymus vulgaris* was found to have the highest antibacterial activity against *Listeria monocytogenes*, *Staphylococcus aureus*, *Salmonella enteritidis*, and *Campylobacter jejuni*, among other EOs. In contrast, *Salvia officinalis* EO was observed to be ineffective for controlling the tested microorganisms.

While cinnamon and mustard EOs were effective to inhibit *Staphylococcus aureus*, *Bacillus cereus*, *Escherichia coli* O157:H7, *Pseudomanas aeruginosa*, *Pseudomonas fluorescens*, *Pseudomonas putida*, *Pectobacterium carotovorum*, and *Salmonella enterica* subsp. *enterica* individually, their synergistic action was only found on *Escherichia coli* O157:H7 and *Pseudomanas putida*. The highest antimicrobial activity was observed on mustard EO [31]. In a study performed by our research group, while cardamom, cumin, and dill weed EOs were effective inhibitors individually against *Campylobacter* spp. [44], their mixtures showed the highest antibacterial activity against *Escherichia coli*, *Campylobacter* spp. and *Staphylococcus aureus* [5]. Similarly, the antibacterial activities of cinnamon and clove EO were tested not only individually but also in combination. Clove EO was more effective than cinnamon EO to inhibit microorganisms, which might be a result of its higher eugenol concentration (53.9%). Their combination showed better antibacterial activity against *Staphylococcus aureus*, *Listeria monocytogenes*, *Pseudomanas aeruginosa*, and *Salmonella typhimurium* compared to their individual applications [34]. However, in another study, cinnamon EO had higher antibacterial activity than clove EO against *Escherichia coli*, *Bacillus subtilis*, *Pseudomanas aeruginosa*, and *Staphylococcus aureus* [45]. The different results obtained might be due to the differences in the composition of EOs. Lavandula species are well-known for their wide variety of biological activities. Although it is commonly used in the cosmetic industry, depending on the linalool, 1,8-cineole, and camphor content, the antibacterial activity of *Lavandula angustifolia* EO is also noteworthy. This significant antibacterial activity was proved against *Escherichia coli*, *Staphylococcus aureus*, and *Pseudomanas aeruginosa* [43]. Moreover, the EO from *Lavandula mairei* Humbert that was predominated by carvacrol had different levels of antibacterial activity against *Listeria innocua*, *Listeria monocytogenes*, *Staphylococcus aureus*, *Bacillus subtilis*, *Proteus vulgaris*, and *Pseudomanas aeruginosa* [40]. Peppermint (*Mentha piperita* L.) EO, which was mainly characterized by menthol and menthone, showed significant levels of antibacterial activity against *Staphylococcus aureus*, *Micrococcus flavus*, *Bacillus subtilis*, *Salmonella enteritidis*, and *Staphylococcus epidermidis* [56]. Furthermore, peppermint EO was active against *Escherichia coli* [47].

### 2.2. Antifungal and Antimycotoxigenic Activities of Essential Oils

In addition to increasing fungal infections, mycotoxin contamination has become an important issue in recent years. Both fungal growth and mycotoxin contamination may result in quality and quantity losses as well as health risks. Despite these facts, studies on antifungal and antimycotoxigenic activities of EOs are limited compared to the studies on their antibacterial activities (Table 2 and Table 3).

Our extensive literature search showed that one of the most extensively studied essential oils on antifungal activity was rosemary (*Rosmarinus officinalis*) EO. In these studies, the antifungal activities of rosemary EO against *Fusarium verticillioides* [57], *Fusarium oxysporum* [58,59], and *Fusarium proliferatum* [58] have been reported. Additionally, antifungal activities against *Mucor pusillus* and *Aspergillus oryzae* [59], *Botrytis cinerea* [60], and *Alternaria alternata* [61] were also noteworthy. However, their sensitivities to corresponding EOs were at different levels. For example, although *Aspergillus niger* inhibition was 93% at 20 µg/mL [59], 67% inhibition of *Fusarium verticillioides* was achieved at a higher concentration (600 µg/mL) [57]. On the other hand, the growth of *Botrytis cinerea* was completely inhibited by rosemary EO at 25.6 µg/mL [60]. Moreover, rosemary EO was effective against *Aspergillus niger* [59,62]. In contrast to *Aspergillus niger*, rosemary EO was not effective for controlling *Aspergillus flavus*, *Penicillium miniolateum*, and *Penicillium oxalicum* [63]. While significant levels of antifungal activity of rosemary EO have been shown by the above-mentioned studies, antimycotoxigenic activity is the other biological activity of rosemary EO that should be considered. The antimycotoxigenic activity of rosemary EO was proved against fumonisin B_1_, fumonisin B_2_ [57], and aflatoxin B_1_ [64,65]. While Aflatoxin B_2_ production was inhibited by rosemary EO [65], it was ineffective on the degradation of zearalenone (ZEA) toxin [66].

One of the most commonly investigated EOs for its antifungal and antimycotoxigenic activity was obtained from *Thymus* species. Although different sensitivities against different fungal cultures were exhibited, EOs from different *Thymus* species have a wide spectrum of antifungal activity (Table 2). Many studies about EOs from *Thymus* species indicated that these activities were superior to its thymol and carvacrol contents. The corresponding EO pronounced antifungal activity against *Aspergillus carbonarius* [67], *Aspergillus niger* [68,69,70], *Aspergillus flavus* [67,69], and *Aspergillus parasiticus* [71]. In addition to *Aspergillus* spp., the other most studied molds were *Penicillium* spp. [67,72,73]. Lastly, *Fusarium solani* [68] and *Botrytis cinerea* [73] have also been subjected to studies on *Thymus* species. In these studies, differences in the results were observed for the obtained minimum inhibition concentration (MIC) values. These differences in the antifungal and antimycotoxigenic activities of EOs may originate from differences in geographical location, harvesting season, or part of the plant that was used during EO preparation. The composition of the EO is also assumed as a key factor, which can vary even within the same species. Variations in the profile and content of components resulted in an extensive difference in databases. This situation was demonstrated with a study that was carried out by Mohammadi et al. [74], who have reported different antifungal activities of EOs from different species of the same plant. The remarkable finding according to this group was that while thymol was the major component in the EO from *Thymus kotschyanus*, it was carvacrol in *Thymus daenensis* EO [74]. Studies on the antimycotoxigenic effects of *Thymus* EO were generally focused on aflatoxins. The inhibition of aflatoxin B_1_ [75,76], aflatoxin B_2_ [76], and aflatoxin G_1_ [75] by using EOs has been demonstrated in different studies. It is interesting to notice that in the presence of *Thymus* EO, the production of fumonisin increased, while aflatoxin production decreased by 4% [71].

In a study comparing the thymus (*Thymus capitatus* L.) EO to eucalyptus (*Eucalyptus globulus* L.) and pirul (*Schinus molle* L.) EOs, it was concluded that thymus was the most effective EO against both *Aspergillus parasiticus* and *Fusarium moniliforme* [71]. *Eucalyptus camaldulensis* is one of the most common plant species, and the effectiveness of its EO commonly characterized by its 1,8-cineole content is well established against many molds [120]. EO from species of *Eucalyptus* possessed antifungal activity against *Fusarium* spp. [101,102], *Botrytis cinerea* [101], and *Aspergillus flavus* [101,103]. The antiaflatoxigenic activity of *Eucalyptus globulus* was proven by [103]. In a study conducted with thymus, clove, and eucalyptus EOs, all EOs showed antifungal activity, but considering their activity levels, it was suggested to use especially thymus and clove EOs as an alternative to synthetic fungicides [70]. With respect to the studies in the literature, one of the most widely studied essential oils is clove EO, and its antifungal activity has been attributed to its main component eugenol [121]. Although the inhibition of *Aspergillus niger* was completely achieved at 200 µL/L [70], at 100 µL/L, the conidial germination of *Aspergillus flavus* was inhibited by 87% [122]. The effectivity of clove EO against *Aspergillus oryzae*, *Aspergillus ochraceus* [86], *Fusarium oxysporum* [100], *Penicillium citrinum*, and *Rhizopus nigricans* [117] was also proved. In addition to in vivo applications, in vitro studies also confirmed the antifungal effects of clove EO to prevent gray mold in strawberries [123] and pomegranate fruits [70]. In addition to the antifungal activities of clove EO, its antimycotoxigenic abilities were also investigated by several researchers. The inhibitory effects of clove EO against ochratoxin A [124] and inhibition of fumonisin B_1_ [36] have been reported. Not only clove EO but also *Salvia officinalis*, *Lavandula dentata*, and *Laurus nobili* EOs were observed to have ochratoxin A (OTA) inhibition ability. Within these EOs, *Laurus nobili* EO was the most effective to inhibit *Aspergillus carbonarius*, and it completely inhibited the OTA production [125].

Another EO that has a wide range of antifungal activity was cumin EO. In addition to being one of the most popular spices in the world due to its strong characteristic flavor [126], the EO obtained from cumin has broad use because of its high antimicrobial activity and broad spectrum of antifungal activity. The antifungal activity of cumin EO has been reported against *Aspergillus* spp. [83,92,93,94,95], *Penicillium* spp. [94,95], and *Botrytis cinerea* [96]. In addition to the molds listed above, another mold that was investigated for its sensitivity against *Cuminum cyminum* EO was *Fusarium oxysporum* [95,96]. However, it should also be noted that there are variations between different EOs with respect to the EO concentration required for the complete fungal inhibition. On the other hand, different data obtained from the studies carried out using broth microdilution and macrodilution methods could be attributed to the screening method [93]. Additionally, as already well known, chemical compositions of EOs can vary depending on the part of the plant used to obtain the EO [110], which leads to differences in the biological activities of EOs. On the other hand, the origin of the plant used to obtain the EO also makes a difference. For example, cumin seed EO from Iran was observed to contain α-pinene (29.2%), limonene (21.7%), 1,8-cineole (18.1%), and linalool (10.5%) as the most significant components [92]. However, the most abundant components in cumin seed EO from the same variety, *Cuminum cyminum* L., and extracted with the same hydrodistillation method but obtained from India were cymene (47.08%), gamma-terpinene (19.36%), and cuminaldehyde (14.92%). In addition to the above-mentioned antifungal activities, another important biological activity of cumin EO is the antimycotoxigenic activity (Table 3). In this sense, at a concentration of 0.5 µL/mL *Cuminum cyminum* (L.) seed EO, aflatoxin B_1_ was completely inhibited [95]. Similar to cumin EO, aflatoxin B_1_ was also inhibited by *Piper bettle* L. EO with a remarkable antifungal activity [127]. On the contrary, the application of EO at low concentration may stimulate mycotoxin production. For instance, OTA and aflatoxin B_1_ production were stimulated by *Salvia officinalis* EO [125] and *Piper bettle* L. EO [127] at low concentrations, respectively.

In addition to the widespread use of oregano for aromatic purposes, the medicinal properties of its EO are also important [138]. The antimicrobial activities of *Origanum vulgare* were extensively studied and well documented. Compared to the antibacterial activities, data on their antifungal activities are limited. Different types and varieties of oregano have been the subject of scientific studies. For example, *Origanum vulgare* EO was observed to have an inhibitory effect on *Fusarium verticillioides* [113]. In addition to the commonly known oregano (*Origanum vulgare*), *Poliomintha longiflora* (Mexican oregano) was also reported to be a good antifungal agent, which was attributed to its thymol, carvacrol, and *p*-cymene contents [114]. Velluti et al. [36] outlined that oregano EO is one of the most effective EO against fumonisin B_1_. Similarly, the mechanism of antiaflatoxigenic action was also directly correlated with the aflatoxin biosynthesis pathway [30]. Another effective EO against fumonisin B_1_ and fumonisin B_2_ was *Curcuma longa* (turmeric) EO, and that inhibitory effect was correlated with the inhibition of fungal growth [97]. However, there are also studies in which fungal inhibition and toxin production were independent of each other. For instance, there was no correlation between the antiaflatoxigenic and antifungal activity of *Rosmarinus officinalis* L. EO [65]. Fumonisin B_1_ inactivation was not achieved at a sufficient level by peppermint EO [131]. However, ZEA reduction by mint EO was reported to be in the range of 19.87% to 30.79% [66]. Unlike the toxin inhibition activity of mint EO at moderate levels, it exhibits a wide spectrum of antifungal activity. The antifungal activity of *Mentha piperita* L. EO, which was mainly dominated by menthol [109,139], was generally attributed to their major components. However, the contribution of minor components to these biological activities should also be considered. When the susceptibility of *Aspergillus* species to *Mentha piperita* EO was compared, the lowest MIC values were observed in *Aspergillus fumigatus* and *Aspergillus clavatus* within *Aspergillus flavus*, *Aspergillus fumigatus*, *Aspergillus oryzae*, and *Aspergillus clavatus* [108]. Different susceptibilities of molds to EOs may be linked to the production of enzymes by the fungus that catalyzes oxidation and thus causes inactivation of the oil [140]. Peppermint (*Mentha piperita* L.) EO also had significant levels of antifungal activities against *A. alternaria* and *Penicillium* spp. with an MIC value of 1.50 μg/mL [56].

In a study investigating and comparing the effectiveness of a group of EOs, including spearmint, peppermint, and cinnamon EOs, the most effective one was found to be cinnamon EO against *Penicillium* spp. Moreover, tested *Penicillium* sp. showed considerable antifungal sensitivity to EOs obtained both from the bark and the leaf of cinnamon [85]. Similarly, within several EOs, including *Mentha haplocalyx* (peppermint) and *Cinnamon zeylanicum* (cinnamon), the most effective one was indicated to be cinnamon EO with the lowest MIC values against *Aspergillus flavus*, *Aspergillus ochraceus*, and *Aspergillus niger* [86]. The most effective components of cinnamon (*Cinnamon zeylanicum*) EO due to their biological activities were indicated to be eugenol and cinnamaldehyde [141]. The antifungal activities of cinnamon EO have been determined against several fungi including *Aspergillus flavus* [89], *Aspergillus ochraceus* [83,86], *Aspergillus niger* [86], *Aspergillus oryzae* [86], *A. parasiticus* [89], and *Fusarium proliferatum* [36]. The inhibitory effect of cinnamon EO from *Cinnamomum casia* (cinnamon, 78% e-cinnamaldehyde) was also proved against *Aspergillus carbonarius* [88]. In addition to *Aspergillus* spp., *Fusarium verticilloides* was also investigated in terms of its sensitivity to cinnamon EOs, including different levels of cinnamaldehyde, the main component of cinnamon EO, as 85% and 99%. It was concluded that higher inhibitory effects were observed when the cinnamaldehyde concentration was higher [84]. In addition to the antifungal activities listed above, the antimycotoxigenic activities of cinnamon EO have also been the subject of many studies. Especially, the inhibition of aflatoxin B_1_ [133], fumonisin B_1_ [36,131], ochratoxin A (OTA) [88], ZEA [66,132], and deoxynivalenol (DON) [132] by cinnamon EO were studied.

Similar to cinnamon EO, antimycotoxigenic properties, specifically, the antiaflatoxigenic activities of *Carum carvi* EO (commercially available as caraway EO) are also at promising levels. Its ability of inhibiting aflatoxin B_1_ has been reported by Lasram et al. [129] and Razzaghi-Abyaneh et al. [75]. Indeed, *Carum carvi* EO was also able to inhibit aflatoxin G_1_, and 94.6% inhibition was achieved at a concentration of 1000 μg/mL [75]. Similarly, in another work, aflatoxin B_1_ and aflatoxin G_1_ production were inhibited with the treatment of *Carum carvi* [89]. Although the most abundant component of *Carum carvi* L. EO was carvone with the reported percentages of 50–65% [89], 67.6% [142], and 78.85% [129], in certain cases, the relative ratio of limonene (69.93%) was observed to be much higher than that of carvone (14.65%) [143]. On the other hand, in another study, the main components of *Carum carvi* L. EO were reported as cuminaldehyde (22.08%), γ-terpinene (17.86%), and γ-terpinene-7-al (15.41%) [75]. These obvious differences in the composition of EOs may be due to the type and origin of the plant material [143]. *Carum carvi* seed EO was also active as an antifungal agent against *Penicillium* spp. [80,82], *Aspergillus* spp. [82,83,89], and *Fusarium* spp. [81]. When compared to other EOs, *Carum carvi* EO was more effective than *Ocimum basilicum* L. EO [80] and EOs from *Pelargonium roseum* L. and *Cymbopogon nardus* (L.) Rendle [81].

As another EO, the toxin inhibition potential of *Ocimum species* EO has been reported for aflatoxin B_1_ [111] and fumonisin [110]. Moreover, promising levels of antifungal activity were reported against *Aspergillus flavus*, *Aspergillus fumigatus*, *Aspergillus terreus*, *Alternaria alternata*, *Penicillium italicum*, *Fusarium nivale*, and *Cladosporium* spp. [111,112].

A wide range of EOs has been investigated in different studies (Table 2 and Table 3). In this review, we tried to summarize the results of research studies from the last few years; however, it should also be noted that there are many studies related with the activities of different EOs such as *Foeniculum vulgare* (fennel) [104,105,106], *Cymbopogon citratus* (lemongrass) [98,99], *Brassica nigra* (mustard) [78,79], and *Melaleuca alternifolia* (tea tree) [107,144,145]. Moreover, differences in the fungal cultures, geographical origin, plant parts from which EO was derived, extraction method, and harvesting time results in the formation of a database containing numerous studies.

## 3. Antioxidant Activities of Essential Oils

Antioxidants are substances that neutralize the adverse effects of oxidative stress [146], and they may be either natural or synthetic. Natural antioxidants are generally preferred by consumers by virtue of the potential health risks of synthetic antioxidant consumption [147]. Plants and different plant parts such as flowers, stems, and roots may be the source of natural antioxidants, including polyphenols, carotenoids, and vitamins. The EOs of these plants exhibit antioxidant activity apart from several biological activities such as antimicrobial, anticancer, anti-inflammatory, and anti-aging [148,149].

Recently, many research studies have been carried out about the antioxidant activity of different EOs. The total phenol (TPC), total flavonoid (TFC), total flavonol, phenolic acid, catechin, lignan, and tannin contents of EOs have been the main parameters measured while evaluating the antioxidant properties. There are several methods used to evaluate the antioxidant activity of EOs obtained from different plants; however, differences in these methods may lead to different results that make comparisons difficult, and thus, investigations on the modification and improvement of these methods still continue to provide the most reliable technique [150]. Moreover, as mentioned in the previous sections of this review, there are several parameters affecting EO composition that may also result in different antioxidant activity values.

In a study by Kulisic et al. [151], the antioxidant properties of the oregano EO were determined by using the β-carotene bleaching (BCB), 2,2-diphenyl-1-picrylhydrazyl (DPPH) radical scavenging and thiobarbituric acid reactive species (TBARS) assays. The tested oregano EO exhibited a different antioxidant power. While oregano EO showed low radical scavenging activity by DPPH assay, in another study conducted by Asensio et al. [152], the antioxidant capacities of EOs of four different oregano species from different regions of Argentina (*Origanum x majoricum*, *Origanum vulgare* subsp. *Vulgare*, and *Origanum vulgare* subsp. *hirtum* clones) were investigated by using 2,2-azino-bis (3-ethylbenzothiazoline-6-sulfonic acid) (ABTS), ferric reducing antioxidant power (FRAP), β-carotene bleaching, and oxygen radical absorbance capacity (ORAC) assays, and it was concluded that the geographical difference is a significant factor on the antioxidant activity of studied species. This phenomenon was also reported by several other researchers [153,154,155]. In addition to the geographical origin of the plant, harvesting time is another factor that may affect the antioxidant activity [156]. From this point of view, Ozkan et al. [157] revealed the free radical scavenging activities (IC_50_ = 116.74–132.93 µg/mL) and reducing antioxidant capacities (23.54–31.02 mg ascorbic acid equivalent (AAE)/g EO) of EOs from Turkish oregano (*Origanum onites* L.) seeds differed based on the harvesting time. In the same study, some selected Turkish oregano samples were also found to be rich in potential natural antioxidant components such as rosmarinic acid. Moreover, carvacrol and thymol have also been reported as the main components that are correlated with the antioxidant activity of origanum EO [156,158]. These components are the dominant phenolic compounds present not only in origanum EO but also in EO from *Thymus* species. Several *Thymus* species were found rich in both carvacrol and thymol and observed to have a high antioxidant activity including *Thymus vulgaris* L. [33,52,159], *Thymus capitatus* L. [53], and *Thymus daenensis* [52,160]. On the other hand, although Ali et al. [16] and Zouari et al. [68] reported that these components were either absent or at a low amount in the composition of *Thymus algeriensis* L. Boiss. et Reut, its EO was indicated as an alternative source of natural antioxidants. The part of the plant where EOs are obtained is another important factor determining their chemical composition and antioxidant activity. For example, different parts of cinnamon such as the bark, leaf, flower, and root may be used in order to obtain its EO, which may result with different concentrations of bioactive components and thus antioxidant activities [161]. It should also be considered that there could be different methods applied while obtaining EOs from their sources. Deng et al. [162] worked on the impact of the molecular distillation method on the antioxidant characteristics of cold-pressed *Citrus paradisi* Macf. (grapefruit) EO as measured by DPPH (IC_50_ = 22.06 mg/mL) and ABTS (IC_50_ = 15.72 mg/mL) methods. The results indicated that this technology could be a good alternative to obtain EOs, depending on the usage area, with proper compositions and without an adverse effect on the antioxidant activity.

The antioxidant activities of *Cinnamomum zeylanicum* Blume (cinnamon) EO [45] and *Eugenia caryophyllus* (clove) EO [45,163] were reported to be related with the composition of EOs. Kallel et al. [164] showed that *Cinnamomum zeylanicum* Blume (cinnamon) EO was composed of different monoterpenes (such as α-pinene) and sesquiterpenes, which were responsible for the remarkable antioxidative activity of cinnamon as also observed in the study of Tepe and Ozaslan [165]. In this study, it was shown that the antioxidant activity is the result of not only major components but also of the minors.

The antioxidant activity of oregano EO was correlated with its composition as reported by Kulisic et al. [151]. This correlation was detected not only for oregano EO but also for several other EOs. In a study conducted with the extracts and EOs of costmary (*Tanacetum balsamita* L.) and tansy (*Tanacetum vulgare* L.), it was shown that the antioxidant potentials were associated with the content of phenolic acids such as caffeic, rosmarinic, and ferulic acids [166]. In another study, the antioxidant activities of *Eucalyptus globulus* and *Eucalyptus radiata* EOs were referred to their major components, namely 1,8-cineole and limonene, respectively [167]. Even though the antioxidant activity is generally correlated with the phenolic compounds of the EOs, recent research studies indicated that the relevant antioxidant activity may also be related with the non-phenolic compounds. It has been proven for limonene, linalool, and citral by Baschieri et al. [168], and these non-phenolic compounds have been found significant in terms of their contribution to antioxidant activity.

**Figure 1:**
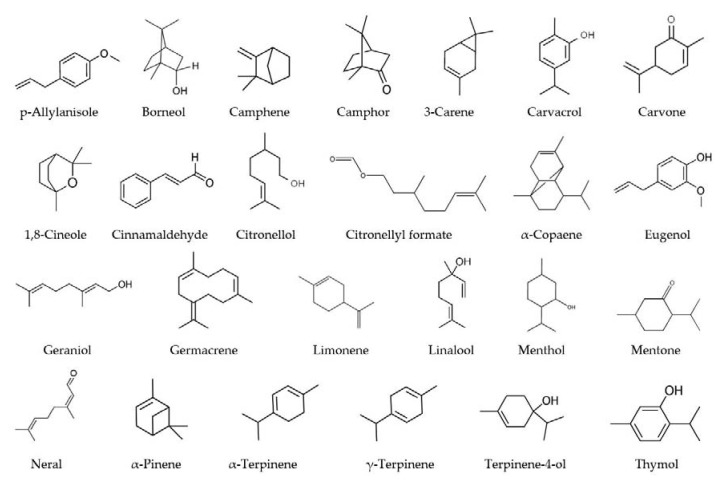
The chemical structures of major active compounds of essential oils (EOs) with antioxidant activity (in alphabetical order).

There are also several studies in which different EOs have been investigated and compared for their antioxidant activity (Table 4). In Figure 1, the chemical structures of some of the main constituents of EOs mentioned in Table 4 are shown. In the study of Purkait et al. [169], the antioxidant activities of EOs from *Piper nigrum* (black pepper), *Cinnamomum zeylanicum* (cinnamon), and *Syzygium aromaticum* (clove) were compared, which also show high antimicrobial activity. DPPH, β-carotene-linoleic acid bleaching, and Fe^2+^ ion chelating methods were carried out to evaluate their antioxidant activities, and the highest activity was observed in *Syzgium aromaticum* EO. Moreover, eugenol (72.46%), cinnamaldehyde (63.82%), and β-caryophyllene (43.47%), which are the main constituents with remarkable amounts in clove, cinnamon, and black pepper EO, respectively were associated with their antioxidant activities. Teixeira et al. [170] examined the antioxidant activity of seventeen different EOs by DPPH and FRAP methods. According to the results of DPPH analysis, only seven of them (*Apium graveolens* (celery seed), *Cymbopogon nardus* (citronella), *Eugenia* spp. (clove), *Thymus capitatus* (origanum), *Petroselinum sativum* (parsley), *Petroselinum sativum* (tarragon), and *Thymus vulgaris* (thyme) EOs) showed observable activity in the range of EC_50_ = 0.04–10.04 mg/mL. Among these, *Eugenia* spp. (clove) and *Thymus capitatus* (origanum) EOs showed 50% inhibition at a lower concentration, which indicated their high antioxidant effect. On the other hand, Stanojevic et al. [171] reported the antioxidant activity of *Aetheroleum basilici* (basil) to be higher than *Aetheroleum menthae piperitae* (peppermint) according to the DPPH assay. In another study, *Mentha viridis* EO was subjected to DPPH, ABTS, and FRAP assays in order to evaluate its antioxidant activity, and the researchers demonstrated the significant biological activities of *Mentha viridis* EO, including antioxidant activity [172].

According to Singh et al. [188], the two major constituents of *Nigella sativa* L. (black cumin) are thmoquinone (37.6%) and *p*-cymene (31.4%). There are studies performed in the past, up until recent years, investigating the antioxidant characteristics of *Nigella sativa* L. (black cumin) [181,188,189,190,191], and in all these reports, the antioxidant activity of *Nigella sativa* L. (black cumin) EO has been mentioned. *Ocimum basilicum* L. (basil) is another plant EO that has been used in recent antioxidant studies [182,192,193]. Ahmed et al. [182] observed that although the EOs of *Ocimum basilicum* L. (basil) collected from several regions have the same three major components—linalool, estragole, and methyl cinnamate—the difference in their percentages were interpreted as a reason for the significant differences in their antioxidant activities.

Citrus is one of the most consumed and harvested fruits all over the World [194,195], and its genus is composed of several types of fruits such as sweet orange, mandarin, grapefruit, lime, and lemon [196]. The antioxidant activity of citrus EOs containing many bioactive compounds is one of the commonly known biological activities [162,197]. Guo et al. [177] examined the antioxidant activities of several citrus EOs and compared their activities based on the results of DPPH and ABTS methods. *Citrus maxima* (honey pomelo) showed the lowest antioxidant activity according to both methods. On the other hand, while *Citrus medica* var. *sarcodactylis Swing* (bergamot) was the EO showing strong antioxidant activity by DPPH assay, *Citrus aurantium* L. (lime) had the highest antioxidant activity results based on the ABTS method. As also mentioned above, it is clear that the antioxidant activity may also vary among EOs depending on the performed analysis method.

Additionally, it has been reported that the combination of EOs showed a synergistic effect on the antioxidant activities of EOs [34,198]. Misharina and Samusenko [199] determined the antioxidant properties of several EOs either as single or in combination (*Citrus limon* L. (lemon), *Citrus paradisi* L. (pink grapefruit), *Coriandrum sativum* L. (coriander), and *Caryophyllus aromaticus* L. (clove buds)). In this study, it has been reported that *Caryophyllus aromaticus* L. (clove buds) had the highest antioxidant activity within the single EOs, and indeed, combinations of EOs generally showed synergistic effects. Different antioxidant activity values of different EO combinations may be correlated with the various major and minor components present in these EOs. There are also several studies in which an isobologram analysis based on the median effect principle (IC_50_) has been used to evaluate the synergistic antioxidant effect of the EOs including the mixture of *Coriandrum sativum* (coriander) and *Cuminum cyminum* (cumin) seed EOs [200] and a combination of *Cinnamomum zeylancium* (cinnamon) and *Syzygium aromaticum* (clove) [34]. In both studies, a synergistic effect on antioxidant activity was observed, and the results were associated with the constituents of EOs.

In addition to the above-mentioned EOs in this review, there are many recent studies performed on the antioxidant activities of different EOs, including *Curcuma longa* L. [97], *Mentha pulegium* L. [180], *Echinophora platyloba* DC. [178], *Artemisia dracunculus* (tarragon) [174], *Pistacia vera* L. [184], *Lavandula angustifolia* Mill. (lavender) and *Lavandula x intermedia* Emeric (lavandin) cultivars [201], *Eucalyptus globulus* and *Eucalyptus radiata* [167], *Tanacetum vulgare* L. [187], *Pelargonium asperum* and *Ormenis mixta* [183], *Agastache foeniculum* [173], *Catha edulis* Forsk cultivars (khat) [176], *Artemisia herba-alba* [175], *Litsea cubeba* [202], *Prangos gaubae* [185], *Psidium cattleianum* Sabine [186], *Pimpinella saxifrage* [11], *Rumex hastatus* D. Don [203], *Laurus nobilis* (laurel), *Zingiber officinale* (ginger) and *Anethum graveolens* (dill) [148], and *Melaleuca alternifolia* [179].

## 4. Recent Trends in Essential Oils

Nowadays, the food industry presents a demand for EOs due to their notable applications as food preservatives [204]. However, their applications in foods are limited because of some distinctive properties such as strong smell, high unpredictability, poor water dissolvability, and instability [19]. Furthermore, while EOs are unstable in the presence of light, heat, oxygen, and humidity, their volatile nature and hydrophobicity restrict their direct use in foods [205,206]. These problems could be solved by enhancing the water solubility and bioavailability, protecting bioactive compounds from extrinsic and intrinsic factors, and removing unpleasant odor and taste in order to use EOs in the food system [207]. For this purpose, novel techniques can be utilized such as encapsulation, edible coating, and active packaging [19]. Encapsulation is a technique that first entraps one component (active agent) into another substance (wall material) and then produces particles in the nanometer (nanoencapsulation) or micrometer (microencapsulation) scale by different techniques [208]. A wide range of strategies could be carried out for the formulation such as polymeric particles, liposomes, solid lipid nanoparticles, liquid crystalline systems, and nanostructured lipid carriers [204,207]. Additionally, nanoemulsion, micro emulsion, nanogel, solid-nano nanoparticles, and liposome methods have been currently used to encapsulate plant bioactive compounds for food preservatives [207]. Different physical, physicochemical, and mechanical methods have been used to encapsulate bioactive compounds. Among them, spray drying, coacervation, emulsification, and ionic gelation are the most commonly used techniques to encapsulate EOs [209]. In line with this, in the last few years, EOs have been incorporated with polymeric matrices to enhance their antifungal activities such as *Eucalyptus staigeriana* [210], *Ocimum sanctum* [211], *Origanum vulgare* [212], cinnamon and lemon grass [213], *Mentha piperita* and *Melaleuca alternifolia* [214].

Nanoscale materials for drug preservation and controlled release such as nanogels have gained attention. Nanogels are preferred because of the features including the effectiveness of bioactive substances at lower concentrations and stability from environmental factors such as ionic strength, pH, light, and temperature [215]. Beyki et al. [18] encapsulated the *Mentha piperita* EO with an encapsulating agent of chitosan and cinnamic acid by nanogel methods. The encapsulated oil showed better antifungal activity under sealed condition, while the free oils were ineffective to completely inhibit *Aspergillus flavus.* Similar to this study, encapsulated *Thymus vulgaris* EO with nanogels consisting of chitosan and benzoic acid was found to be more effective against *Aspergillus flavus*. Based on the volatility and non-stability characteristics of EOs, encapsulation technology was found to be appropriate for increasing the shelf life and improving the antifungal properties according to the study [216].

Chitosan biopolymer is generally recognized as safe due to its non-toxicity, biocompatibility, and biodegradability. It has gained a great deal of attention in last few years as an encapsulation wall material because of some of its properties such as being insecticidal, antimicrobial, antioxidant, and having film-forming properties [217,218,219,220]. Therefore, chitosan has been used to encapsulate EOs such as clove [221], *Cuminum cyminum* [215], *Bunium persicum* [222], and *Foeniculum vulgare* [217]. Singh et al. [211] found that while chitosan-encapsulated *Ocimum sanctum* EO inhibited the growth of *Aspergillus flavus* and aflatoxin B_1_ secretion at 60 and 20 μL/L, respectively, unencapsulated EO had the similar activity at 300 and 200 μL/L. The encapsulated *Ocimum sanctum* EO had two times higher radical scavenging activity than unencapsulated EO. In addition to its antioxidant activity, the phenolic content increased with encapsulation, and the increase in phenolic content resulted in the prolonged shelf life of stored herbal raw material. Moreover, the nanoencapsulation of EOs such as *Thymus zygis* [223] and *Thymus vulgaris* [216] enhanced the antifungal activity against *Alternaria alternata* and *Aspergillus flavus*, respectively, when compared with free EOs. The activity of nanoencapsulation has been studied not only under in vitro conditions but also under in vivo conditions such as on food products. Gonçalves et al. [224] studied the encapsulated *Thymus vulgaris* EO as a natural preservative in food products and showed that encapsulated thyme EO increased the induction time of oxidation, the cake shelf life up to 30 days, as well as the antimicrobial activity. Zein-encapsulated *Thymus vulgaris* and *Origanum vulgare* EOs showed higher antimicrobial and antioxidant activities. Moreover, tested EOs protected from thermal degradation at baking processes [12].

In addition to nanoencapsulation, there are also several microencapsulation studies of EOs [225,226]. For instance, the morphological and sensorial properties of Jujube (*Ziziphus jujuba* Mill) fruit with microencapsulated *Zingiber officinale* (ginger) EO in chitosan and sodium carboxymethyl cellulose were enhanced while maintaining the nutritional value [227]. On the surface of untreated jujube fruits, severe blackspots were observed after 7 days of storage; however, no rotten jujube fruits were observed in samples with microencapsulated EOs. Red and decay index were measured for the evaluation of freshness and the sensory properties of jujube fruits. An increment of red index, which is the degree of maturity, was restricted with EO microencapsulation. Sensory evaluations of EO microencapsulated fruit samples were carried out by 10 panelists. For appearance, crunchiness, firmness, and juiciness, they reported that EO microencapsulated fruits had better sensory quality characteristics [227]. In another study, ripening in *Syringe* EO microencapsulated (SEOM) *Prunus persia* fruit has been delayed. Additionally, ethylene production was lower than the control during the storage period. SEOM application resulted in an increase in the peach-like aroma and decrease in the grass-like aroma, mainly in the last period of storage, protecting the peach aroma during cold storage [228]. The use of cyclodextrin as an encapsulating agent in microencapsulation is also recommended due to its unique advantages including heat and oxidative stability [226]. Generally, during storage the color of vegetables may change. With EO microencapsulation, there was no significant difference in the color of lettuce compared to the untreated sample during the entire storage period. However, L* and (-a*/b*) scores of samples treated with free EO were observed to decrease compared to the untreated sample during storage. It was also found that microencapsulated beta cyclodextrin complexes with thyme EO showed higher antimicrobial activity and protected the EO from degradation. Additionally, antimicrobial activity of this complex was observed during the storage of pork meat system [229].

Currently, the reasons such as increasing interest in the quality of food products by consumers and preferring those that are packed with environmentally friendly material have increased the interest in new packaging materials. As a result, new types of edible films produced by using food-grade compounds can be used as primary packaging material, which is developed to extend the shelf life of food products [230,231,232]. However, these days, the commercial use of edible films is significantly restricted because of cost disadvantages. In addition to the cost problems, difficulty in the production process and the strictness of the regulation both restrict the use of edible films and coatings [232]. Edible films that are generally made from single or combinations of polysaccharides, lipids, and proteins obtain advantages such as water, oxygen, and aroma barrier properties with improving the food appearance and quality at all stages of the food processing [1,230]. The features of the film are actually directly related to the edible compound. In this manner, compared to protein films, while chitosan films showed better oil barrier properties, their water vapor properties were lower [231]. As is commonly known, EOs could be used as natural antioxidant and antimicrobial agents instead of synthetic ones in food products. However, their dominant flavor causes limitations on their use. In respect to this, the addition of EOs to edible coating on food packages provides several advantages such as increasing the film performance by eliminating some limitations [10,231]. From the antimicrobial perspective, the incorporation of EOs as antimicrobial agents directly into food packaging systems is a form of active packaging [233].

Cheese samples were coated with sodium alginate solutions containing 1%, 2%, and 3% *Pimpinella saxifraga* EO by dipping into sodium alginate and EO solutions for 2 min at room temperature. In the study, an acute toxicity test was conducted to evalute the use of EOs for food safety purposes with a mice model. According to the results, there was no harmful effect at 250 and 500 mg/kg; however, 750 and 1000 mg/kg concentrations resulted in some abnormal behaviour. On the other hand, the consumer acceptance of this new active edible coating was evaluated by 21 panelists using a five-point hedonic scale. The coated samples were more appreciated in terms of odor, flavor, and color without any change in the texture of the product [11]. In another study, beef samples were coated by immersing to the cinnamon EO-loaded *Shahri Balangu* seed mucilage (SBM) solution for 1 min. Coated beef possessed several benefits in terms of better texture, reduced lipid oxidation, and total viable count. Moreover, the sensory evaluation of beef samples was also conducted with well-trained panelists by using a nine-point hedonic scale. The colors of uncoated and SBM-coated beef samples were unacceptable at the end of 9 days of storage; however, beef coated with EO-loaded SBM was found to be acceptable. In addition, while the shelf-lives of uncoated and SBM coated beef were 6 days, EO-loaded SBM coated beef had a shelf-life of 9 days [10].

Mahcene et al. [234] evaluated the incorporation of EOs in sodium alginate-based edible film on an active food packaging system. Edible films were prepared by the blending of 2.5% sodium alginate film-forming solution and dispersing the EOs in the presence of Tween 80. While *Artemisia herba alba* EO incorporation improved the thermal properties, it showed the lowest peroxide value (2.58 meq O_2_/kg) on the packaged sunflower oil in contrast to normal packaged sunflower oil (4.719 meq O_2_/kg). Furthermore, while EO-incorporated film had antimicrobial activity against both Gram-positive and Gram-negative bacteria, the highest antioxidant capacity was observed for *Ocimum basilicum* EO film with 23% in comparison with *Mentha pulegium* EO film’s 4%. It can be understood from that study that sodium alginate edible film incorporated with EOs is an alternative for protecting food quality with increasing shelf life. In another study carried out with carbohydrate-based films, enriched EOs revealed that films with cinnamon EO had lower antioxidant activity than pure cinnamon EO due to the loss of cinnamon EO during film preparation, drying, and storage [235].

Acosta et al. [236] studied the antifungal activity and film properties of cinnamon bark, clove, and oregano EO incorporated on starch gelatin films. EOs had no significant impact on water vapor and oxygen permeability; however, they increased the transparency of the films. The three films containing EO inhibited the growth of *Fusarium oxysporum* and *Colletotrichum gloeosporioides* due to their phenolic compounds: eugenol, carvacrol, and thymol. However, cinnamon bark EO films were recommended to protect the spoilage from *Fusarium oxysporum*.

Chitosan is widely used as a coating agent on edible films. It has remarkable antimicrobial activity against a variety of fungi, Gram-positive, and Gram-negative bacteria [237]. Polyvinyl alcohol/gum arabic/chitosan (PVA/GA/CS) composite films due to their hydrophilic nature showed high degree of swelling. This can be overcome by the incorporation with EOs such as ginger and black pepper oil; with this application, swelling properties can decrease because of their high hydrophobic nature [238]. Similarly, chitosan films incorporated with turmeric EO also reduced film solubility and swelling. In addition to these advantages, it also showed antiaflatoxigenic activity [237].

Composite films containing ginger and black pepper EOs decreased the water solubility and were more resistant to breakage with improved heat stability than PVA/GA/CS composite films [238]. Moreover, carboxymethyl cellulose–polyvinyl alcohol films enriched with cinnamon EO showed a lower transmittance value than pure films. This was a significant property because high UV absorbance on food packaging restricts the lipid oxidation on food [235]. Moreover, chitosan coating with nanoencapsulated *Satureja khuzestanica* EO retarded the microbial growth and chemical spoilage during the meat product storage period [239]. Alginate-based edible coating enriched with EO constituents (eugenol and cinnamaldehyde) also retarded microbial spoilage by preserving the nutritional and sensory attributes of *Arbutus unedo* L. fresh fruit during storage [240].

To summarize, EOs could be directly used in food products with some novel applications such as encapsulation, edible films, and edible coatings. Encapsulation technology provides improvements on oxidation stability, heat stability, and the antimicrobial and antioxidant activity of EOs. Moreover, the addition of EOs to edible films and coatings can increase heat stability and resistance to breakage, reduce swelling and solubility, and also add to and/or improve the antimicrobial and antioxidant activities of the products.

## 5. Conclusions and Future Aspects

Essential oils and their components are important because of their low cost, availability, and wide range of biological activities. Another advantage is that when they are used in appropriate proportions, they do not disturb the taste and aroma and thus improve the shelf-life of the food material. While antibacterial and antioxidant abilities of EOs are well documented, studies on antifungal and antimycotoxigenic activities are still limited. From the health and economical aspects, it is essential to find effective, safe, and economical antifungal agents to control both the growth and mycotoxin production of fungi. However, different results have been observed in different studies as a result of the differences in fungal cultures used during the analysis of antimicrobiological activity, geographical origin, harvesting time, part of the plant from which EO was derived, and extraction and analysis methods, and it is of critical importance to consider these parameters while working with EOs, since they affect their composition, profile, and biological activities.

The use of essential oil mixtures that are designed in accordance with the characteristics of the food can be considered as a new perspective in terms of the organoleptic properties of the food. Due to the instability of EOs under environmental stresses such as temperature and light, novel technologies might be helpful to protect and improve their characteristics and biological activities. On the other hand, further studies should focus on the synergistic effects between different essential oils and/or different components, along with their action of mechanisms. Another recommendation is that besides these investigations against monocultures, it is also necessary to investigate the antifungal actions against polycultures. Lastly, new strategies for improving the stability of essential oils and decreasing the required concentration for ensuring food safety with minimal sensorial changes can be an interesting research area for researchers.

## Figures and Tables

**Table 1:** Overview of studies about antibacterial properties of essential oils.

Essential Oil From	Bacterial Culture	Method	MIC ^1^	Reference
*Artemisia herba alba*	*Escherichia coli*, *Pseudomanas aeruginosa*, *Staphylococcus aureus*	Broth dilution	4–42.67 µL/mL	[43]
*Anethum graveolens* (dill weed)	*Escherichia coli*, *Staphylococcus aureus*	Broth microdilution	3.75 µL/mL	[5]
*Anethum graveolens* (dill weed)	*Camplylobacter coli*, *Campylobacter jejuni*	Broth microdilution	0.012–0.025 µL/mL	[44]
*Backhousia citriodora* (lemon myrtle)	*Escherichia coli*, *Listeria monocytogenes*, *Staphylococcus aureus*	Broth microdilution	0.16–0.62 (%)	[41]
*Brassica* spp. (mustard)	*Bacillus cereus*, *Escherichia coli*, *Escherichia coli* O157:H7, *Pseudomanas aeruginosa*, *Pseudomonas fluorescens*, *Pseudomonas putida*, *Pectobacterium carotovorum*, *Salmonella enterica* subsp. *enterica*, *Staphylococcus aureus*	Broth dilution	12.5–200 µg/mL	[31]
*Cinnamomum camphora* (camphor)	*Bacillus subtilis*, *Escherichia coli*, *Staphylococcus aureus*, *Salmonella typhimurium*	Microdilution broth	2–4 mg/mL	[38]
*Cinnamomum zeylanicum* (cinnamon)	*Bacillus subtilis*, *Escherichia coli*, *Staphylococcus aureus*, *Salmonella typhimurium*	Microdilution broth	0.12–0.25 mg/mL	[38]
*Cinnamomum zeylanicum* (cinnamon)	*Bacillus cereus*, *Escherichia coli*, *Escherichia coli* O157:H7, *Pseudomanas aeruginosa*, *Pseudomonas fluorescens*, *Pseudomonas putida*, *Pectobacterium carotovorum*, *Salmonella enterica* subsp. *enterica*, *Staphylococcus aureus*	Broth dilution	100–400 µg/mL	[31]
*Cinnamomum zeylanicum* (cinnamon)	*Bacillus cereus*, *Escherichia coli*, *Listeria monocytogenes*, *Staphylococcus aureus*, *Pseudomanas aeruginosa*, *Salmonella typhimurium*	Microdilution	72.27–114.63 µL/mL	[34]
*Cinnamomum zeylanicum*	*Bacillus subtilis*, *Escherichia coli*, *Pseudomanas aeruginosa*, *Pseudomonas putida*, *Staphylococcus aureus*,	Broth macrodilution	1.25 µL/mL	[45]
*Cinnamomum zeylanicum*	*Listeria monocytogenes*, *Staphylococcus aureus*, *Salmonella enteritidis*, *Campylobacter jejuni*	Broth dilution	0.06–7.25 µL/mL	[46]
*Cuminum cyminum* (cumin)	*Escherichia coli*, *Staphylococcus aureus*	Broth microdilution	3.75–15 µL/mL	[5]
*Cuminum cyminum* (cumin)	*Camplylobacter coli*, *Campylobacter jejuni*	Broth microdilution	0.05 µL/mL	[44]
*Cymbopogon citrus*	*Escherichia coli*, *Staphylococcus aureus*	Dilution	6.25 µL/mL	[47]
*Cymbopogon nardus* (citronella)	*Bacillus subtilis*, *Escherichia coli*, *Staphylococcus aureus*, *Salmonella typhimurium*	Microdilution broth	2–4 mg/mL	[38]
*Cymbopogon winterianus* (lemon)	*Escherichia coli*, *Enterococcus faecalis*, *Klebsiella pneumoniae*, *Staphylococcus aureus*	Microdilution	4.03–8.37 mg/mL	[48]
*Elettaria cardamomum* (cardamom)	*Escherichia coli*, *Staphylococcus aureus*	Broth microdilution	3.75–7.50 µL/mL	[5]
*Elettaria cardamomum* (cardamom)	*Camplylobacter coli*, *Campylobacter jejuni*	Broth microdilution	0.025 µL/mL	[44]
*Eugenia caryophyllus*	*Bacillus subtilis*, *Escherichia coli*, *Pseudomanas aeruginosa*, *Pseudomanas putida*, *Staphylococcus aureus*	Broth macrodilution	1.25–10 µL/mL	[45]
*Lavandula angustifolia*	*Escherichia coli*, *Pseudomanas aeruginosa*, *Staphylococcus aureus*	Broth dilution	1.33–42.67 µL/mL	[43]
*Lavandula angustifolia*	*Escherichia coli*, *Staphylococcus aureus*	Dilution	125–250 µL/mL	[47]
*Lavandula mairei* Humbert	*Bacillus subtilis*, *Listeria innocua*, *Listeria monocytogenes*, *Proteus vulgaris*, *Pseudomanas aeruginosa*, *Staphylococcus aureus*	Broth macrodilution	0.6–1.2 mg/mL	[40]
*Melaleuca alternifolia Cheel* (tea tree)	*Escherichia coli*, *Enterococcus faecalis*, *Klebsiella pneumoniae*, *Staphylococcus aureus*	Microdilution	0.55–17.6 mg/mL	[48]
*Mentha haplocalyx* (peppermint)	*Bacillus subtilis*, *Escherichia coli*, *Staphylococcus aureus*, *Salmonella typhimurium*	Microdilution broth	2–4 mg/mL	[38]
*Mentha piperita*	*Escherichia coli*, *Staphylococcus aureus*	Dilution	62.5 µL/mL	[47]
*Mentha pulegium*	*Bacillus subtilis*, *Escherichia coli*, *Listeria* *monocytogenes*, *Pseudomanas aeruginosa*, *Proteus mirabilis*, *Staphylococcus aureus*	Broth microdilution	0.25 to >2 mg/mL	[49]
*Origanum vulgare* (oregano)	*Listeria monocytogenes*, *Staphylococcus aureus*, *Salmonella enteritidis*, *Campylobacter jejuni*	Broth dilution	0.016–1 µL/mL	[46]
*Origanum vulgare* (oregano)	*Bacillus cereus*, *Staphylococcus aureus*, *Salmonella enteritidis*, *Salmonella typhimurium*	Broth dilution	160–640 µg/mL	[50]
*Pimpinella anisum* (anise)	*Bacillus subtilis*, *Escherichia coli*, *Staphylococcus aureus*, *Salmonella typhimurium*	Microdilution broth	0.5–2 mg/mL	[38]
*Pimpinella saxifraga*	*Bacillus cereus*, *Escherichia coli*, *Listeria monocytogenes*, *Micrococcus luteus*, *Pseudomanas aeruginosa*, *Salmonella typhimurium*	Microdilution	0.78–3.12 mg/mL	[11]
*Piger nigerium* (black pepper)	*Bacillus cereus*, *Escherichia coli*, *Listeria monocytogenes*, *Staphylococcus aureus*, *Pseudomanas aeruginosa*, *Salmonella typhimurium*	Microdilution	81.64–124.47 µL/mL	[34]
*Rosmarinus officinalis*	*Bacillus subtilis*, *Escherichia coli Listeria monocytogenes*, *Pseudomanas aeruginosa*, *Proteus mirabilis*, *Staphylococcus aureus*	Broth microdilution	0.5 to >2 mg/mL	[49]
*Rosmarinus officinalis* L. (rosemary)	*Escherichia coli*, *Enterococcus faecalis*, *Klebsiella pneumoniae*, *Staphylococcus aureus*	Microdilution	0.67–10.8 mg/mL	[48]
*Rosmarinus officinalis*	*Listeria monocytogenes*, *Staphylococcus aureus*, *Salmonella enteritidis*, *Campylobacter jejuni*	Broth dilution	0.5–85 µL/mL	[46]
*Salvia officinalis*	*Listeria monocytogenes*, *Staphylococcus aureus*, *Salmonella enteritidis*, *Campylobacter jejuni*	Broth dilution	1.56–60 µL/mL	[46]
*Salvia sclarea* L. (clary sage)	*Escherichia coli*, *Enterococcus faecalis*, *Klebsiella pneumoniae*, *Staphylococcus aureus*	Microdilution	1.38–44.23 mg/mL	[48]
*Satureja hortensis* L.	*Escherichia coli*, *Listeria monocytogenes*, *Staphylococcus aureus*, *Salmonella typhimurium*, *Pseudomanas aeruginosa*	Agar dilution	2–4 mg/mL	[39]
*Syzygium aromaticum* (clove)	*Bacillus subtilis*, *Escherichia coli*, *Staphylococcus aureus*, *Salmonella typhimurium*	Microdilution broth	0.5–1 mg/mL	[38]
*Syzygium aromaticum* (clove)	*Escherichia coli*, *Enterococcus faecalis*, *Klebsiella pneumoniae*, *Staphylococcus aureus*	Microdilution	0.21 mg/mL	[48]
*Syzygium aromaticum* (clove)	*Escherichia coli*, *Listeria monocytogenes*, *Staphylococcus aureus*, *Salmonella typhimurium*	Dilution	0.304 mg/mL	[51]
*Syzygium aromaticum* (clove)	*Bacillus cereus*, *Escherichia coli*, *Listeria monocytogenes*, *Staphylococcus aureus*, *Pseudomanas aeruginosa*, *Salmonella typhimurium*	Microdilution	58.54–85.67 µL/mL	[34]
*Thymus algeriensis*	*Bacillus cereus*, *Escherichia coli*, *Listeria monocytogenes*, *Staphylococcus aureus*, *Pseudomanas aeruginosa*	Macrobroth dilution	1–4.5 µL/mL	[16]
*Thymus daenensis*	*Bacillus cereus*, *Escherichia coli*, *Staphylococcus aureus*, *Salmonella typhimurium*	Dilution	20 µg/mL	[52]
*Thymus vulgaris*	*Bacillus cereus*, *Escherichia coli*, *Staphylococcus aureus*, *Salmonella typhimurium*	Dilution	20 µg/mL	[52]
*Thymus vulgaris*	*Bacillus cereus*, *Staphylococcus aureus*, *Salmonella enteritidis*, *Salmonella typhimurium*	Broth dilution	320–640 µg/mL	[50]
*Thymus vulgaris*	*Listeria monocytogenes*, *Staphylococcus aureus*, *Salmonella enteritidis*, *Campylobacter jejuni*	Broth dilution	0.12–0.25 µL/mL	[46]
*Zanthoxylum bungeanum* (pepper)	*Bacillus subtilis*, *Escherichia coli*, *Staphylococcus aureus*, *Salmonella typhimurium*	Microdilution broth	1–4 mg/mL	[38]
*Zataria multiflora*	*Bacillus cereus*, *Escherichia coli*, *Staphylococcus aureus*, *Salmonella typhimurium*	Dilution	5–10 µg/mL	[52]
*Zingiber officinale Roscoe* (ginger)	*Escherichia coli*, *Enterococcus faecalis*, *Klebsiella pneumoniae*, *Staphylococcus aureus*	Microdilution	0.15–9.85 mg/mL	[48]

^1^ MIC: minimum inhibition concentration.

**Table 2:** Overview of studies about antifungal properties of essential oils.

Essential Oil From	Fungal Culture	Method	MIC/Inhibition ^1^	Reference
*Anacyclus valentinus*	*Aspergillus flavus*, *Aspergillus parasiticus*, *Aspergillus ochraceus*, *Penicillium expansum*, *Penicillium citrinum*, *Fusarium graminearum*, *Fusarium moniliforme*	Macrodilution	1.25–2.5 µL/mL	[77]
*Brassica nigra* (mustard)	*Aspergillus fumigatus*, *Aspergillus nomius*, *Aspergillus niger*, *Penicillium cinnamopurpureum*, *Penicillium expansum*, *Penicillium viridicatum*	Vapor diffusion	0.012–0.06 µg/mL_air_	[78]
*Brassica sp.* (mustard)	*Penicillium roqueforti*, *Penicillium verrucosum*, *Fusarium oxysporum*, *Penicillium expansum*, *Aspergillus niger*, *Botryotinia fuckeliana*, *Aspergillus flavus*, *Geotrichum spp.*, *Aspergillus ochraceus*, *Rhizopus stolonifer*	Broth macrodilution	0.8–50 µg/mL	[79]
*Bupleurum falcatum*	*Aspergillus flavus*, *Alternaria alternata*, *Fusarium oxysporum*	Broth microdilution	0.5–2 µg/mL	[74]
*Carum Carvi* (caraway)	*Penicillium carneum*, *Penicillium cavernicola*, *Penicillium aurantiogriseum*, *Penicillium nalgiovense*, *Penicillium polonicum*, *Mucor racemosus*	Agar dilution	0.7–1.5 µL/mL	[80]
*Carum carvi* L.	*Fusarium oxysporum*, *Fusarium verticillioides*, *Penicillium brevicompactum*, *Penicillium expansum*, *Aspergillus flavus*, *Aspergillus fumigatus*	Agar dilution	1–3.6 µL/mL	[81]
*Carum carvi*	*Aspergillus flavus*, *Botrytis cinerea*, *Penicillium italicum*, *Penicillium expansum*, *Penicillium commune*, *Rhizopus stolonifer*, *Rhizopus lyococcus*	Agar overlay technique	100% inhibiton at 500 ppm	[82]
*Carum* spp. (caraway)	*Aspergillus ochraceus*	Macrodilution	0.625 μL /mL	[83]
*Cinnamon* spp. (cinnamon)	*Aspergillus ochraceus*	Macrodilution	0.078 μL /mL	[83]
*Cinnamon* sp. (cinnamon)	*Fusarium verticilloides*	Semisolid agar antifungal susceptibility technique	60 μL /L	[84]
*Cinnamon* sp. (cinnamon)	*Penicillium* spp., *Clodosporium* spp.	Disc diffusion	100% inhibiton at 20 µL	[85]
*Cinnamomum camphora* (camphor)	*Aspergillus niger*, *Aspergillus ochraceus*, *Aspergillus oryzae*	Gradient plate	2 mg/mL	[86]
*Cinnamomum casia*	*Aspergillus flavus*	Broth microdilution	62.5 µg/mL	[87]
*Cinnamomum casia* (cinnamon)	*Aspergillus carbonarius*	Poisoned food technique	100% inhibition at 50–100 µL/L	[88]
*Cinnamomum cassia*	*Aspergillus flavus*, *Aspergillus carbonarius*, *Penicillium viridacatum*	Inhibition zone method	1.67, >5 µL/mL	[67]
*Cinnamomum zeylanicum* (cinnamon)	*Aspergillus niger*, *Aspergillus ochraceus*, *Aspergillus oryzae*	Gradient plate	0.062–0.125 mg/mL	[86]
*Cinnamomum zeylanicum*	*Botrytis cinerea*, *Penicillium expansum*	Broth microdilution	625–1250 µg/mL	[73]
*Cinnamomum zeylanicum* (cinnamon)	*Aspergillus flavus*, *Aspergillus parasiticus*	Microatmosphere	100% inhibition at 500 µL/L	[89]
*Citrus aurantifolia* (mirim lime)	*Botrytis cinerea*, *Penicillium digitatum*, *Trichoderma viride*	Microdilution	625 to >2500 µg/mL	[90]
*Citrus latifolia* (tahiti lime)	*Botrytis cinerea*, *Penicillium digitatum*, *Trichoderma viride*	Microdilution	625 to >2500 µg/mL	[90]
*Citrus limon* L. (lemon)	*Aspergillus parasiticus*	Agar dilution	≥1500 ppm	[91]
*Citrus limon* (siciliano lemon)	*Botrytis cinerea*, *Penicillium digitatum*, *Trichoderma viride*	Microdilution	312 to >2500 µg/mL	[90]
*Citrus limonia* (cravo lime)	*Botrytis cinerea*, *Penicillium digitatum*, *Trichoderma viride*	Microdilution	312 to >2500 µg/mL	[90]
*Cuminum* sp.	*Aspergillus ochraceus*	Macrodilution	2.5 μL/mL	[83]
*Cuminum cyminum* L. (cumin)	*Aspergillus flavus*, *Aspergillus parasiticus*, *Aspergillus niger*	Broth dilution	750–1000 ppm	[92]
*Cuminum cyminum* L.	*Aspergillus flavus*, *Aspergillus fumigatus*	Broth microdilution Broth macrodilution	1.5 mg/mL 0.25 mg/mL	[93]
*Cuminum cyminum*	*Aspergillus flavus*, *Aspergillus fumigates*, *Aspergillus niger*, *Aspergillus ochraceus*, *Penicillium citrinum*, *Penicillium chrysogenum*, *Fusarium verticillioides*	Broth microdilution	1000–2000 μg/mL	[94]
*Cuminum cyminum*	*Aspergillus flavus*	Poisoned food technique	0.6 μL /mL	[95]
*Cuminum cyminum*	*Fusarium oxysporum*, *Rhodotorula glutinis*, *Botyrtis cinerea*	Disc diffusion	80.9–91.4% inhibition at 10 µL	[96]
*Curcuma longa* (turmeric)	*Fusarium verticillioides*	Broth dilution	73.7 μg/mL	[97]
*Cymbopogon citrati* (lemon grass)	*Aspergillus flavus*, *Aspergillus ochraceus*, *Aspergillus parasiticus*, *Aspergillus westerdijkiae*	Gas diffusion	15.625 µL/L_air_	[98]
*Cymbopogon citratus* (lemon grass)	*Aspergillus parasiticus*, *Aspergillus flavus*, *Aspergillus clavatus*	Vapor phase	96% inhibition at 500 µL/L_air_	[99]
*Cymbopogon citratus* (lemon grass)	*Fusarium oxysporum*	Broth microdilution	31.25 ppm	[100]
*Cymbopogon martini*	*Aspergillus flavus*, *Aspergillus carbonarius*, *Penicillium viridacatum*	Inhibition zone method	1.67, >5 µL/mL	[67]
*Cymbopogon nardus*	*Fusarium oxysporum*, *Fusarium verticillioides*, *Penicillium brevicompactum*, *Penicillium expansum*, *Aspergillus flavus*, *Aspergillus fumigatus.*	Agar dilution	0.6–6.7 µL/mL	[81]
*Cymbopogon nardus* (citronella)	*Aspergillus niger*, *Aspergillus ochraceus*, *Aspergillus oryzae*	Gradient plate	1–2 mg/mL	[86]
*Eucalyptus* sp.	*Fusarium gramineraum*, *Fusarium asiaticum*, *Fusarium verticillioides*, *Fusarium oxysporum*, *Aspergillus flavus*, *Botyrtis cinerea*	Poisoned food technique	33–75% inhibition at 1000 µL/L	[101]
*Eucalyptus camaldulensis*	*Fusarium oxysporum*, *Fusarium proliferatum*, *Fusarium soloni*, *Fusarium subglutinans*, *Fusarium verticillioides*	Poisoned food technique	7–8 µL/mL	[102]
*Eucalyptus globulus*	*Aspergillus parasiticus*, *Fusarium moniliforme*	Disc diffusion	9–27% inhibition at 500 µL/L	[71]
*Eucalyptus globulus*	*Aspergillus flavus*, *Aspergillus parasiticus*	Contact and volatile assay	100% inhibition at 500 µL	[103]
*Foeniculum vulgare* (bitter fennel)	*Fusarium oxysporum*, *Fusarium profileratum*, *Fusarium verticillioides*	Modified microdilution	3.25–10 mg/mL	[104]
*Foeniculum vulgare* (fennel)	*Aspergillus flavus *	Microdilution broth	10 µg/mL	[105]
*Foeniculum vulgare*	*Colletotrichum gloeosporioides *, *Phytophthora capsici*, *Sclerotinia sclerotiorum*, *Fusarium fujikuroi*	Agar disc diffusion	1.5 to >2 µL/mL	[106]
*Melaleuca alternifolia* (tea tree)	*Botrytis cinerea*, *Penicillium expansum*	Agar dilution	2–6 mL/L	[107]
*Mentha haplocalyx* (peppermint)	*Aspergillus niger*, *Aspergillus ochraceus*, *Aspergillus oryzae*	Gradient plate method	1–2 mg/mL	[86]
*Mentha piperita* (mint)	*Fusarium oxysporum*	Broth microdilution	125 ppm	[100]
*Mentha piperita* L. (peppermint)	*Aspergillus clavatus*, *Aspergillus flavus*, *Aspergillus fumigatus*, *Aspergillus fumigates*, *Aspergillus oryzae*	Broth microdilution	0.5–4 µL/mL	[108]
*Mentha piperita* L. (peppermint)	*Alternaria alternaria*, *Aspergillus flavus*, *Aspergillus fumigates*, *Aspergillus variecolor*, *Fusarium acuminatum*, *Fusarium solani*, *Fusarium oxysporum*, *Fusarium tabacinum*, *Moliniana fructicola*, *Rhizoctomia saloni*, *Sclorotinia minör*, *Sclorotinia selerotiorum*, *Trichophyton mentagrophytes*	Microdilution	0.5–10 µg/mL	[56]
*Mentha piperita* L.	*Aspergillus flavus*, *Aspergillus glaucus*, *Aspergillus niger*, *Aspergillus ochraceous*, *Colletotrichum gloesporioides*, *Colletotrichum musae*, *Fusarium oxysporum*, *Fusarium semitectum*	Poisoned food technique	90–100% inhibition at 0.2% EO	[109]
*Ocimum basilicum*	*Fusarium verticillioides*	Modified semisolid agar antifungal susceptibility	1–2 μL/mL	[110]
*Ocimum basilicum* L. (basil)	*Mucor racemosus*, *Penicillium aurantiogriseum*, *Penicillium carneum*, *Penicillium cavernicola*, *Penicillium nalgiovense*, *Penicillium polonicum*	Agar dilution	4.5–9 µL/mL	[80]
*Ocimum gratissium*	*Aspergillus flavus*	Broth dilution	0.6–0.7 µL/mL	[111]
*Ocimum gratissium*	*Fusarium verticillioides*	Modified semisolid agar antifungal susceptibility	0.3–5 μL/mL	[110]
*Ocimum sanctum*	*Aspergillus flavus*	Poisoned food technique	0.3 µL/mL	[112]
*Origanum majorana* (marjoram)	*Botrytis cinerea*, *Penicillium expansum*	Broth microdilution	5000–10,000 µg/mL	[73]
*Origanum x majoricum*	*Aspergillus flavus *, *Penicillium oxalicum*, *Penicillium minioluteum*	Poisoned food technique	400–550 ppm	[63]
*Origanum vulgare* L. (oregano)	*Fusarium verticillioides*	- ^2^	250 µL/L	[113]
*Origanum vulgare* spp. *hirtum*	*Aspergillus flavus *, *Penicillium oxalicum*, *Penicillium minioluteum*	Poisoned food technique	350–650 ppm	[63]
*Origanum vulgare* L. sspp. *vulgare*	*Aspergillus flavus *, *Penicillium oxalicum*, *Penicillium minioluteum*	Poisoned food technique	200–550 ppm	[63]
*Pelargonium roseum* L.	*Fusarium oxysporum*, *Fusarium verticillioides*, *Penicillium brevicompactum*, *Penicillium expansum*, *Aspergillus flavus*, *Aspergillus fumigatus.*	Agar dilution	0.8–5.1 µL/mL	[81]
*Pimpinella anisum* (anise)	*Aspergillus niger*, *Aspergillus ochraceus*, *Aspergillus oryzae*	Gradient plate	0.5–1 mg/mL	[86]
*Poliomintha longiflora* (mexican oregano)	*Aspergillus flavus*, *Botrytis cinerea*, *Colletotrichum gloeosporioides*, *Penicillium expansum*	Agar dilution	0.8–1.4 g/L	[114]
*Poliomintha longiflora* (mexican oregano)	*Aspergillus flavus*, *Botrytis cinerea*, *Colletotrichum gloeosporioides*, *Penicillium expansum*	Agar dilution	0.8–1.4 g/L	[114]
*Rosmarinus officinalis* L.	*Fusarium oxysporum*, *Fusarium proliferatum*	Disc diffusion	50% inhibition at 1122–1641 μL/L	[58]
*Rosmarinus officinalis* L. (rosemary)	*Aspergillus niger*, *Aspergillus oryzae*, *Fusarium oxysporum*, *Mucor pusillus*	Disc diffusion	93–100% inhibition at 20 μg/mL	[59]
*Rosmarinus officinalis* L. (rosemary)	*Aspergillus flavus*, *Aspergillus niger*	Broth macrodilution	1 μL/mL	[62]
*Rosmarinus officinalis*	*Aspergillus flavus*	Macrodilution	500 µg/mL	[65]
*Rosmarinus officinalis* (rosemary)	*Aspergillus niger*	Microdilution	1000 µg/mL	[115]
*Rosmarinus officinalis* L.	*Botrytis cinerea*	Volatile phase assay	100% inhibition at 1.6 μg/mL_air_	[60]
*Rosmarinus officinalis* (rosemary)	*Fusarium verticillioides*	Microdilution	150 µg/mL	[57]
*Rosmarinus officinalis* (rosemary)	*Alternaria alternata*	Microdilution	1000 µg/mL	[61]
*Rosmarinus officinalis* (rosemary)	*Botrytis cinerea*, *Penicillium expansum*	Broth microdilution	2500–5000 µg/mL	[73]
*Satureja khusiztanica*	*Aspergillus niger*, *Botrytis cinerea*, *Penicillium digitatum*, *Rhizopus stolonifer*	Broth macrodilution	600–1200 µL/L	[116]
*Schinus molle* (pirul)	*Aspergillus parasiticus*, *Fusarium moniliforme*	Disc diffusion	4.4–15.3% inhibition at 500 µL/L	[71]
*Stachys pubescens*	*Aspergillus flavus *, *Alternaria alternata*, *Fusarium oxysporum*	Broth microdilution	0.5–1 µg/mL	[74]
*Syzgium* sp. (clove)	*Aspergillus niger*	Contact assay	100% inhibition at 200 µL/L	[70]
*Syzgium aromaticum* (clove)	*Aspergillus niger*, *Aspergillus ochraceus*, *Aspergillus oryzae*	Gradient plate	0.25 mg/mL	[86]
*Syzygium aromaticum * (clove)	*Fusarium oxysporum*	Broth microdilution	31.25 ppm	[100]
*Syzygium aromaticum * (clove)	*Aspergillus flavus*, *Aspergillus parasiticus*	Microatmosphere method	100% inhibition at 500 µL/L	[89]
*Syzygium aromaticum * (clove)	*Aspergillus flavus*, *Penicillium citrinum*, *Rhizopus nigricans*,	Agar dilution method	25–50 µL/mL	[117]
*Thymus algeriensis*	*Aspergillus niger*, *Fusarium soloni*	-	1–2 µL/mL	[68]
*Thymus broussonnetii* subs. *hannonis*	*G. citri-aurantii*, *Penicillium digitatum*, *Penicillium italicum*	Agar dilution	>4000 µL/L	[72]
*Thymus capitatus*	*Aspergillus parasiticus*, *Fusarium moniliforme*	Disc diffusion	77.7–91.2% inhibition at 500 µL/L	[71]
*Thymus daenensis*	*Aspergillus flavus *, *Alternaria alternata*, *Fusarium oxysporum*	Broth microdilution	1–4 µg/mL	[74]
*Thymus kotschyanus*	*Aspergillus flavus *, *Alternaria alternata*, *Fusarium oxysporum*	Broth microdilution	0.5–1 µg/mL	[74]
*Thymus leptobotyrs*	*Geotrichum citri-aurantii*, *Penicillium digitatum*, *Penicillium italicum*	Agar dilution	<500 µL/L	[72]
*Thymus mongolicus Ronn*	*Aspergillus flavus*, *Aspergillus carbonarius*, *Penicillium viridacatum*	Inhibition zone method	2.33, >5 µL/mL	[67]
*Thymus riatarum*	*Geotrichum citri-aurantii*, *Penicillium digitatum*, *Penicillium italicum*	Agar dilution	<500–1000 µL/L	[72]
*Thymus satureidos* subsp. *pseudomastichina*	*Geotrichum citri-aurantii*, *Penicillium digitatum*, *Penicillium italicum*	Agar dilution	<500–1000 µL/L	[72]
*Thymus* spp.	*Aspergillus niger*	Contact assay	100% inhibition at 200 µL/L	[70]
*Thymus villosus*	*Aspergillus flavus*, *Aspergillus fumigatus*, *Aspergillus niger*	Broth macrodilution	0.32–2.5 µL/mL	[69]
*Thymus vulgaris*	*Aspergillus flavus*, *Aspergillus fumigatus*, *Fusarium oxysporum*, *Fusarium verticillioides*, *Penicillium expansum*, *Penicillium brevicompactum*	Agar dilution	0.8–2.3 µL/mL	[81]
*Thymus vulgaris* L. (thyme)	*Aspergillus parasiticus*	Agar dilution	2500 ppm	[91]
*Thymus vulgaris* (thyme)	*Botrytis cinerea*, *Penicillium expansum*	Broth microdilution	312–625 µg/mL	[73]
*Thymus zygis* subsp.	*Aspergillus niger*, *Aspergillus flavus*, *Aspergillus fumigatus*	Macrodilution	0.16–0.64 µL/mL	[118]
*Xylopia aethiopica*	*Aspergillus flavus*, *Aspergillus fumigatus*, *Aspergillus niger*, *Aspergillus versicolor*, *Fusarium oxysporum*	Incorporation	3000–4000 ppm	[119]
*Zingiber officinale*	*Aspergillus flavus*, *Aspergillus fumigates*, *Aspergillus niger*, *Aspergillus ochraceus*, *Penicillium citrinum*, *Penicillium chrysogenum*, *Fusarium verticillioides*	Microdilution	1250–2500 μg/mL	[94]

^1^: The inhibition (%) was stated for studies in which the MIC value is not indicated, ^2^: It is not specified.

**Table 3:** Overview of studies about the antimycotoxigenic properties of essential oils.

Essential Oil From	EO Concentration	Mycotoxin	Mycotoxin Inhibition (%)	Method	Reference
*Ageratum conyzoides* L.	0.2–0.5 mg/mL	AF ^1^ B1 AF B2 AF G1	6.88–84.1 58.73–85.71 61.11–96.3	LC-MS-MS, LOD ^2^: NI ^3^	[128]
*Carum carvi* L. (caraway)	0.1–0.3%	AF B1	49.4–99.6	HPLC, LOD: 2 ng/g LOQ ^4^: 5 ng/g	[129]
*Carum carvi* L. (caraway)	10–1000 µg/mL	AF B1 AF G1	1.1–80 35.4–94.6	HPLC, LOD: NI	[75]
*Carum carvi*	500 µl/L_air_	AF B1 AF B2	100	TLC, LOD: NI	[89]
*Carum copticum*	1000 µg/mL 10–1000 µg/mL	AF B1 AF G1	100 23.22–100	TLC, LOD: NI	[130]
*Cinnamon*	140 µg/ml	Fum B1	66.65	ELISA	[131]
*Cinnamon*	500 µg/g	DON ^5^ ZEA ^6^	100	HPLC, LOD: NI	[132]
*Cinnamon*	210–280 µg/mL	Fum ^7^ B1	88–93.35	ELISA, LOD: NI	[84]
*Cinnamomum casia*	50–75 µl/L	OTA ^8^	58–90	HPLC, LOD: 1 ng/g	[88]
*Cinnamomum jensenianum* Hand.-Mazz	1–8 µL/mL	AF B1	31.6–100	TLC-UV, LOD: NI	[133]
*Cinnamomum zeylanicum*, Sri Lanka (cinnamon leaf)	100–200 µL/mL	DON	8.08–13.74	HPLC, LOD: NI	[134]
*Cinnamomum zeylanicum*, Sri Lanka (cinnamon leaf)	100–200 µL/mL	ZEA	13.23–16.87	HPLC, LOD: 0.01 μg/mL	[66]
*Cinnamomum zeylanicum*, Indonesia (cinnamon bark)	100–200 µL/mL	DON	41.55–46.92	HPLC, LOD: NI	[134]
*Cinnamomum zeylanicum*, Indonesia (cinnamon bark)	100–200 µL/mL	ZEA	79.79–89.29	HPLC, LOD: 0.01 μg/mL	[66]
*Cinnamomum jensenianum* Hand.-Mazz	1–8 µL/mL	AF B1	31.6–100	TLC-UV, LOD: NI	[133]
*Citrus aurantifolia* (sour lime)	10–1000 µg/mL	AF B1 AF G1	5.5–89.6 26.9–89.2	HPLC, LOD: NI	[75]
*Citrus grandis* (white grapefruit)	100–200 µg/mL	DON	29.05–35.05	HPLC, LOD: NI	[134]
*Citrus grandis* (white grapefruit)	100–200 µL/mL	ZEA	15.15–70.81	HPLC, LOD: 0.01 μg/mL	[66]
*Citrus limonum* (lemon leaf)	100–200 µL/mL	DON	57.10–62.73	HPLC, LOD: NI	[134]
*Citrus limonum* (lemon leaf)	100–200 µL/mL	ZEA	26.97–66.56	HPLC, LOD: 0.01 μg/mL	[66]
*Citrus paradisi* (pink grapefruit)	100–200 µL/mL	DON	46.01–52.48	HPLC, LOD: NI	[134]
*Citrus paradisi* (pink grapefruit)	100–200 µL/mL	ZEA	1.61–5.05	HPLC, LOD: 0.01 μg/mL	[66]
*Coriandrum sativum* L. (coriander)	0.1–0.7%	AF B1	45.6–100	HPLC, LOD: 2 ng/g LOQ: 5 ng/g	[129]
*Cuminum cyminum* seed	0.1–0.5 µL/mL	AF B1	17.9–93.4	TLC, LOD: NI	[95]
*Curcuma longa* L.	17.9–294.9 µg/mL	Fum B1	33.05–99.11	HPLC; LOD: 0.125 ng/L LOQ: 0.312 ng/L	[97]
*Curcuma longa* L.	17.9–294.9 µg/mL	Fum B2	30–99.4	HPLC; LOD: 0.125 ng/L LOQ: 0.312 ng/L	[97]
*Cymbopogon martinii* (palmarosa)	100–200 µL/mL	DON	59.95–72.18	HPLC, LOD: NI	[134]
*Cymbopogon martinii* (palmarosa)	100–200 µL/mL	ZEA	80–87.27	HPLC, LOD: 0.01 μg/mL	[66]
*Eucalyptus radiata* (eucalyptus leaf oil)	100–200 µL/mL	DON	37.47–37.70	HPLC, LOD: NI	[134]
*Eucalyptus radiata* (eucalyptus leaf oil)	100–200 µL/mL	ZEA	38.48–41.01	HPLC, LOD: 0.01 μg/mL	[66]
*Laurus nobilis*	0.1–0.2%	OTA	80.92–97.32	HPLC, LOD: 0.3 ng OTA/mL, LOQ: 0.5 ng OTA/mL	[125]
*Lavandula dentata*	0.1%	OTA	92.06	HPLC, LOD: 0.3 ng OTA/mL, LOQ: 0.5 ng OTA/mL	[125]
*Lippia turbinata* var. *integrifolia* (Griseb.) (poleo)	2000–3000 µL/L	OTA	18.1–100	HPLC, LOD: 1 ng/g	[124]
*Mentha* sp. (mint)	100 µL/mL	ZEA	19.87–30.79	HPLC, LOD: 0.01 μg/mL	[66]
*Mentha spicata* L. (spearmint)	0.1–0.9 µL/mL	AF B1	9.28–100	TLC, LOD: NI	[135]
*Ocimum gratissimum*	0.1–0.5 µL/mL	AF B1	36.7–100	TLC, LOD: NI	[111]
*Ocimum sanctum*	0.1–0.2 µL/mL	AF B1	82.43–100	Broth culture technique LOD: NI	[112]
*Pëumus boldus* Mol. (boldo)	1000–2000 µL/L	OTA	1.6–100	HPLC, LOD: 1 ng/g	[124]
*Piper bettle*	0.2–0.5 µL/mL	AF B1	15–84.6	TLC	[127]
*Plectranthus amboinicus* (Indian borage)	100–500 ppm	OTA	26.08–100	HPLC	[136]
*Rosmarinus officinalis* L.	75–600 μg/mL	Fum B1 Fum B2	0–99.6 0–99.4	HPLC, LOD: 0.125 ng/L LOQ: 0.312 ng/L	[57]
*Rosmarinus officinalis* L.	75–600 μg/mL	Fum B1 Fum B2	0–99.6 0–99.4	HPLC, LOD: 0.125 ng/L LOQ: 0.312 ng/L	[57]
*Rosmarinus officinalis* L.	250–450 ppm	AF	1.87–100	TLC, LOD: NI	[64]
*Rosmarinus officinalis* L.	100–250 µg/mL	AF B1 AF B2	63.1–100 82.3–100	HPLC, LOD: 0.5 ng/mL	[65]
*Rosmarinus officinalis* (rosemary)	100 µL/mL	ZEA	19.71–22.32	HPLC, LOD: 0.01 μg/mL	[66]
*Salvia officinalis*	0.3–0.5%	OTA	97.68–97.89	HPLC, LOD: 0.3 ng OTA/mL, LOQ: 0.5 ng OTA/mL	[125]
*Syzygium aromaticum* L. (clove)	1000–5000 µL/L	OTA	64.6–100	HPLC, LOD: 1 ng/g	[124]
*Thymus capitatus*	0.1 g/mL	AF B1 Fum B1	23.3 −53	HPLC, LOD: NI	[71]
*Thymus vulgaris* (thyme)	10–1000 µg/mL	AF B1 AF G1	22.1–100 49.5–100	HPLC, LOD: NI	[75]
*Thymus vulgaris*	150 µg/mL	AF B1 AF B2	100 100	HPLC, LOD: 333 ng/mL LOQ: 1000 ng/mL	[76]
*Zataria multiflora* Boiss.	100–200 ppm	Citrinin	68.86–92.44	HPLC (RP-HPLC) LOD: 0.9 × 10^−7^ M	[137]

^1^: Aflatoxin, ^2^: Limit of detection, ^3^: No information provided, ^4^: Limit of quantificatiom, ^5^: Deoxynivalenol, ^6^: Zearalenone, ^7^: Fumonisin, ^8^: Ochratoxin A.

**Table 4:** Overview of studies about antioxidant properties of essential oils.

Essential Oil From	Most Abundant Compounds ^1^	Method	Results	Reference
*Aetheroleum basilici* (basil)	Linalool (39.9%), E-anethol (31.5%), longifolene (4.9%), eugenol (4.8%), α-terpinyl acetate (3.1%)	DPPH	EC_50_ = 0.002–0.494 mg/mL	[171]
*Aetheroleum menthae piperitae* (peppermint)	Menthol (45.4%), menthone (24.4%), iso-menthone (8.3%), menthyl acetate (6%), 1,8-cineole (5.5%)	DPPH	EC_50_ = 58.41–n.d. ^2^ mg/mL	[171]
*Agastache foeniculum*	Methyl chavicol (83.1%), limonene (3.4%), spathulenol (3.1%), caryophyllene oxide (3.1%), β-gurjunene (1.7%)	DPPH ABTS	30.8–93.5% (1–10 mg/mL EO) 44.3–92.1% (1–10 mg/mL EO)	[173]
*Anethum graveolens * (dill)	Neral (27%), carvone (25.7%), limonene (20.6%), dill apiole (8%), *trans*-dihydrocarvone (4.9%)	DPPH Ferrous reducing power β-carotene-linoleic acid assay Superoxide anion scavenging	IC_50_ = 3000 μg/mL EC_50_ = 2400 μg/mL 4000 μg/mL 400 μg/mL	[148]
*Artemisia dracunculus* (tarragon)	*p*-Allylanisole (84.03%), ocimene (E)-β (7.46%), ocimene (Z)-β (6.24%), limonene (1.42%)	DPPH TPC TFC Flavonol content	IC_50_ = 65.4 μg/mL 24.10 mg GAE ^3^/g dry sample 20 mg QE ^4^/g dry sample 14.5 mg/g dry sample	[174]
*Artemisia herba-alba*	β-Thujone (41.9%), α-thujone (18.4%), camphor (13.2%), germacrene D (4.8%), 1,8-cineole (3.4%)	DPPH Chelating assay β-carotene assay FRAP	IC_50_ = 5030 μg/mL IC_50_ = 2300 μg/mL IC_50_ = 159 μg/mL IC_50_ = 79 μg/mL	[175]
*Catha edulis* Forsk cultivars (khat)	Limonene (30-n.d.%), tritetracontane (12-n.d.%), 1-phenyl-1,2-propanedione (11.6-n.d.%), 1-hydroxy,1-phenyl-2-propanone (8.1–1.9%), *o*-mentha-1(7),8-dien-3-ol (8.5-n.d.%)	DPPH	29.1–29.5% (23.5–23.6 μg AAE/kg of fresh khat sample)	[176]
*Cinnamomum zeylanicum* Blume (cinnamon)	Cinnamaldehyde (77.34%), *trans*-cinnamyl acetate (4.98%), 1,4-benzenedicarboxylic acid (3.55%), 1,8-cineole (3.19%), α-pinene (2.6%)	Phosphomolybdenum assay DPPH H_2_O_2_^5^	108.75 mg of EO/equivalent to 1 mg of vitamin C in terms of antioxidant power 21.3% inhibition 55.2% inhibition	[164]
*Cinnamomum zeylanicum* Blume (cinnamon)	*(E)*-Cinnamaldehyde (81.39%), *(E)*-cinnamyl acetate (4.2%), *(Z)*-cinnamaldehyde (3.42%), 1,8-cineole (1.9%), dihydrocinnamaldehyde (1.85%)	Phosphomolybdenum assay CUPRAC FRAP DPPH ABTS	111.46 mg TEs ^6^/g sample 9.82 mg TEs/g sample 3.98 mg TEs/g sample 3.49% inhibition (0.30 mg TEs/g sample) 19.20% inhibition (1.03 mg TEs/g sample)	[165]
*Cinnamomum zeylanicum* (cinnamon)	Cinnamaldehyde (68.2%), eugenol (9.57%), β-caryophyllene (7.21%), 1,2-benzenedicarboxylic acid, mono(2-ethylhexyl) ester (3.27%)	DPPH β-carotene linoleic acid bleaching assay Ferrous (Fe^2+^) ion chelating efficacy	4.62–57.56% inhibition Lower inhibitory activity than clove and black pepper 2.13–43.86% activity	[169]
*Citrus aurantium* L. (lime)	d-Limonene (61.85%), γ-terpinene (9.15%), linalool (8.52%), octanal (5.28%), α-pinene (3.02%)	ABTS DPPH	89.74% inhibition 34.25%	[177]
*Citrus limon Burm* F. (lemon)	d-Limonene (61.72%), α-pinene (13.97%), 3-carene (13.67%), citral (1.88%), geranial (1.29%)	ABTS DPPH	41.57% inhibition 32.85%	[177]
*Citrus maxima* (honey pomelo)	d-Limonene (46.36%), myrcene (16.09%), *cis*-*p*-mentha-2,8-dien-1-ol (2.68%), β-pinene (2.41%), *cis*-linaloloxide (2.38%)	ABTS DPPH	11.64% inhibition 6.40%	[177]
*Citrus. medica var. sarcodactylis* Swin (bergamot)	d-Limonene (48.94%), α-pinene (2.88%), cis-carveol (2.49%), myrcene (2.29%), nootkatone (1.95%)	ABTS DPPH	74.71% inhibition 77.2%	[177]
*Citrus sinensis (Lour.) Osbe* (sweet orange)	d-Limonene (79.28%), 3-carene (7.76%) α-pinene (2.28%), linalool (1.66%), sabinene (1.32%)	ABTS DPPH	40.71% inhibition 25.34%	[177]
*Curcuma longa* (turmeric)	α-Turmerone (42.6%), β-turmerone (16.0%), ar-turmerone (12.9%), α-phellandrene (6.5%), 1,8-cineole (3.2%)	ABTS DPPH	0.54 mg/mL 10.03 mg/mL	[97]
*Echinophora platyloba DC.*	Linalool (16.02%), *trans*-β-ocimene (11.58%), α-pinene (7.10%), anisole, 2,4,6-trimethyl (6.98%), spathulenol (5.29%)	DPPH	IC_50_ = 122.62 µg/ml	[178]
*Eucalyptus globulus*	1,8-Cineole (eucalyptol) (63.81%), α-pinene (16.06%), aromadendrene (3.68%), *o*-cymene (2.35%)	DPPH β-carotene bleaching	IC_50_ = 2.9 *v*/*v* IC_50_ = 2.72 *v*/*v*	[167]
*Eucalyptus radiata*	Limonene (68.51%), α-terpineol (8.6%), α-terpinyl acetate (6.07%), α-pinene (3.01%), terpinen-4-ol (1.61%)	DPPH β-carotene bleaching	IC_50_ = 4.56 *v*/*v* IC_50_ = 6.54 *v*/*v*	[167]
*Laurus nobilis* (laurel)	1,8-Cineole (56%), α-terpinyl acetate (9%), 4-terpineol (5.2%), α-terpineol (4.7%), α-pinene (3.8%), linalool (3.8%)	DPPH Ferrous reducing power β-carotene-linoleic acid assay Superoxide anion scavenging	IC_50_ = 135 μg/mL EC_50_ = 1850 μg/mL 3600 μg/mL 610 μg/mL	[148]
*Melaleuca alternifolia*	Terpinene-4-ol (31.11%), γ-terpinene (25.30%), α-terpinene (12.7%), 1,8-cineole (6.83%), *p*-cymene (4.23%)	DPPH Hydroxyl radical scavenging activity TBARS method	EC_50_ = 48.35 μg/mL EC_50_ = 43.71 μg/mL IC_50_ = 135.9 μg/mL	[179]
*Mentha pulegium* L.	Pulegone (70.66%), neo-menthol (11.21%), menthone (2.63%), *cis*-isopulegone (2.33%), piperitenone (1.58%)	DPPH	IC_50_ = 69.60 μg/mL	[180]
*Mentha viridis*	Carvone (37.26%), 1.8-cineole (11.82%), terpinen-4-ol (8.72%), limonene (5.27%), campher (4.31%)	DPPH ABTS FRAP	IC_50_ = 80.45 μg/mL IC_50_ = 139.59 μg/mL IC_50_ = 101.78 μg/mL	[172]
*Nigella sativa* L. (black cumin)	*p*-Cymene (36.2%), thymoquinone (11.27%), α-thujone (10.03%), longifolene (6.32%), β-pinene (3.33%)	DPPH	82.1–92.1%	[181]
*Ocimum basilicum* L. (sweet basil) from Assiut, Minia and BeniSuef	Linalool (31.65%), estragole (17.37%), methyl cinnamate (15.14%), bicyclosesquiphellandrene (6.01%), eucalyptol (4.04%)	DPPH	IC_50_ = 11.23–55.15 mg/mL	[182]
*Origanum x majoricum* from different provinces of Argentina	*trans*-Sabinene hydrate (28.1–24.3%), thymol (16.9–12.1%), terpinen 4 ol (11.1–6.6%), γ-terpinene (7.5–7%), *orto*-cymene (7.8–2.2%)	ABTS FRAP BCB ORAC	0.163 mM Trolox/mg of essential oils 0.072 mM ascorbic acid/mg of oil 89.2% (from Neuquén) 1.024–1.281 TE	[152]
*Origanum vulgare* subsp. *hirtum clone* from different provinces of Argentina	*trans*-Sabinene hydrate (22.9–17.9%), thymol (18.6–17.1%), terpinen 4 ol (9.5–6.2%), γ-terpinene (8–7.1%), *orto*-cymene (6.3–5.1%)	ABTS FRAP BCB ORAC	0.210 mM Trolox/mg of essential oils 0.185 mM ascorbic acid/mg of oil 75.3% 1.064–1.393 TE	[152]
*Origanum vulgare* subsp. *vulgare* from different provinces of Argentina	*trans*-Sabinene hydrate (27.2–23.4%), thymol (17.2–14.4%), terpinen 4 ol (11–7.8%), γ-terpinene (9.8–7.3%), *orto*-cymene (5.6–2.3%)	ABTS FRAP BCB ORAC	0.206 mM Trolox/mg of essential oils 0.173 mM ascorbic acid/mg of oil 79.3% (from Rio Negro) 1.155–1.708 TE	[152]
*Ormenis mixta*	Germacrene (11.46%), 1,8 cineol (10.29%), *cis*-methyl isoeugenol (9.04%), butyric acid (8.54%), δ-elemene (5.46%)	DPPH	IC_50_ = 0.59 mg/mL	[183]
*Pelargonium asperum*	Citronellol (25.07%), citronellyl formate (10.53%), geraniol (10.46%), buthyl anthranilate (5.94%), isomenthone (5.88%)	DPPH	IC_50_ = 14.62 mg/mL	[183]
*Pimpinella saxifraga*	Anethole (59.47%), pseudoisoeugenol (20.15), *p*-anisaldehyde (7.53%), thellungianin G (6.17%), 4,11-selinadiene (2.99%)	DPPH FRAP	IC_50_ = 6.81 µg/mL EC_50_ = 35.2 µg/mL	[11]
*Piper nigrum* (black pepper)	β-Caryophyllene (43.47%), caryophyllene oxide (14.64%), octadecanoic acid (5.26%), n-hexadecanoic acid (4.45%), humulene (3.86%)	DPPH β-carotene linoleic acid bleaching assay Ferrous (Fe^2+^) ion chelating efficacy	11.24–64.46% inhibition Medium inhibitory activity between clove and cinnamon 6.64–62.48% activity	[169]
*Pistacia vera* L. variety Bronte (pistachio hull)	4-Carene (31.74%), α-pinene (23.58%), D-limonene (8%), δ-3-carene (7.73%), camphene (4.13%)	FRAP DPPH	IC_50_ = 0.063 mg/mL IC_50_ = 0.878 mg/mL	[184]
*Prangos gaubae*	Germacrene D (26.7%), caryophyllene oxide (14.3%), (E)-caryophyllene (13.8%), spathulenol (11.3%), limonene (2.8%)	ABTS FRAP	2.02 mmol TEs/g sample 0.37 mmol TEs/g sample	[185]
*Psidium cattleianum * Sabine	α-Copaene (21.96%), eucalyptol (15.05%), δ-cadinene (9.63%), β-selinene (7.73%), α-selinene (6.42%)	DPPH	16.19–4.01% (50–100 mg/mL EO concentration)	[186]
*Syzygium aromaticum * (clove)	Eugenol (72.46%), eugenyl acetate (4.18%), β-caryophyllene (3.73%), tau muurolol (2.83%), isoeugenol (2.12%)	DPPH β-carotene linoleic acid bleaching assay Ferrous (Fe^2+^) ion chelating efficacy	29.36–77.28% inhibition Higher inhibitory activity 9.56–72.68% activity	[169]
*Tanacetum balsamita * L. (costmary)	β-Thujone (84.43%), α-thujone (4.68%), eucalyptol (4.07%), thymol (0.67%), β-eudesmol (0.64%)	DPPH FRAP	13.59 µmol Trolox/g 339.1 µmol Trolox/g	[166]
*Tanacetum vulgare* L. (tansy)	*trans*-Chrysanthenyl acetate (18.39%), β-thujone (14.28%), (E)-dihydrocarvone (11.02%), artemisia ketone (9.15%), *cis*-chrysanthenol (3.93%)	DPPH FRAP	13.86 µmol Trolox/g 585.6 µmol Trolox/g	[166]
*Tanacetum vulgare* L.	Camphor (30.48%), borneol (14.8%), 1,8-cineole (10.8%), camphene (7.29%), bornyl acetate (5.53%)	DCFH-DA ^7^	IC_50_ = 51 μg/mL	[187]
*Thymus capitatus* L. (thymus)	Thymol (51.22%), carvacrol (12.59%), γ-Terpinene (10.3%), trans-13-Octadecenoic acid (9.04%), linalool (2.29%)	DPPH Ferric reducing power Phosphomolybdenum assay	IC_50_ = 0.619 µg/mL EC_50_ = 2.13 µg/mL EC_50_ = 0.78 µg/mL	[53]
*Thymus daenensis* Celak	Thymol (70.12%), *p*-cymene (5.12%), carvacrol (4.99%), carvone (3.12%), borneol (2.96%)	DPPH Phosphomolybdate assay	IC_50_ = 0.26 mg/mL 1.59 mg of AAE/g of dry weight	[52]
*Thymus kotschyanus* Celak (thymus)	Carvacrol (27.8%), thymol (16.8%), carvacrol acetate (6.87%), phytol (6.8%), thymoquinone (5.4%)	DPPH Phosphomolybdate assay	IC_50_ = 0.16 mg/mL 2.78 mg of AAE/g of dry weight	[52]
*Thymus vulgaris* L.	Thymol (25.78%), carvacrol (17.47%), thymoquinone (7.11%), eugenol (6.36%), β-pinene (6.31%)	DPPH Phosphomolybdate assay	IC_50_ = 0.3 mg/mL 2.01 mg of AAE/g of dry weight	[52]
*Zataria multiflora*	Carvacrol (46.23–39.14%), thymol (18.8–14.82%), thymol acetate (5.72–2.25%), eugenol (5.15-n.d.%), carvacrol acetate (4.92–1.21%)	Phosphomolybdate assay	1.96–2.41 mg of AAE/g of dry weight	[52]
*Zingiber officinale * (ginger)	Camphene (11.5%), β -phellandrene (10.7%), 1,8-cineole (10.4%), α-zingiberene (6.9%), borneol (6.4%)	DPPH Ferrous reducing power β-carotene-linoleic acid assay	IC_50_ = 470 μg/mL EC_50_ = 1900 μg/mL 1900 μg/mL	[148]

^1^ Five most abundant compounds (>1%), ^2^: not determined, ^3^: gallic acid equivalent, ^4^: quercetin equivalent, ^5^: hydrogen peroxide radical scavenging assay, ^6^: Trolox equivalents, ^7^: dichloro-dihydro-fluorescein diacetate assay.
